# Magneto-optical investigation of spin–orbit torques in metallic and insulating magnetic heterostructures

**DOI:** 10.1038/ncomms9958

**Published:** 2015-12-08

**Authors:** Mohammad Montazeri, Pramey Upadhyaya, Mehmet C. Onbasli, Guoqiang Yu, Kin L. Wong, Murong Lang, Yabin Fan, Xiang Li, Pedram Khalili Amiri, Robert N. Schwartz, Caroline A. Ross, Kang L. Wang

**Affiliations:** 1 Department of Electrical Engineering, Device Research Laboratory, University of California, Los Angeles, California 90095, USA; 2 Departmentof Material Science and Engineering, Massachusetts Institute of Technology, Cambridge, Massachusetts 02139, USA

## Abstract

Manipulating magnetism by electric current is of great interest for both fundamental and technological reasons. Much effort has been dedicated to spin–orbit torques (SOTs) in metallic structures, while quantitative investigation of analogous phenomena in magnetic insulators remains challenging due to their low electrical conductivity. Here we address this challenge by exploiting the interaction of light with magnetic order, to directly measure SOTs in both metallic and insulating structures. The equivalency of optical and transport measurements is established by investigating a heavy-metal/ferromagnetic-metal device (Ta/CoFeB/MgO). Subsequently, SOTs are measured optically in the contrasting case of a magnetic-insulator/heavy-metal (YIG/Pt) heterostructure, where analogous transport measurements are not viable. We observe a large anti-damping torque in the YIG/Pt system, revealing its promise for spintronic device applications. Moreover, our results demonstrate that SOT physics is directly accessible by optical means in a range of materials, where transport measurements may not be possible.

## Abstract (web summary)

The study of spin orbit torques in insulating materials via conventional transport methods is restricted due to low electrical conductivity. Here, the authors use magneto-optical methods to measure spin orbit torques in ferromagnetic-insulator/heavy-metal heterostructures.

## Graphical abstract

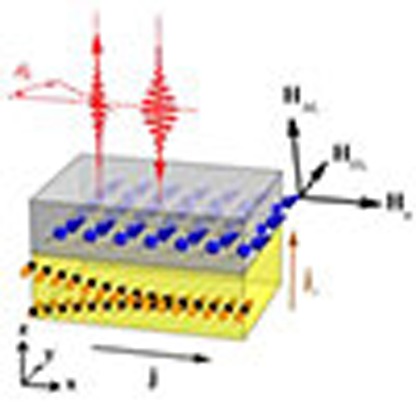

Current-induced manipulation of magnetic order through spin–orbit torques (SOTs) has attracted much attention, with the potential of enabling novel spintronic devices for memory and logic applications^1^,^2^,^3^,^4^,^5^,^6^,^7^,^8^,^9^,^10^,^11^,^12^,^13^,^14^. Specifically, metallic magnets incorporating high spin–orbit elements have been used to realize magnetic memory devices with fast switching and ultralow power consumption^6^,^8^,^9^,^10^. Beyond metallic structures, interests in magnetic insulators and controlling their dynamics by SOTs have been rising due to the inherently zero charge current and low energy dissipation of these materials^15^,^16^,^17^,^18^,^19^,^20^,^21^,^22^,^23^,^24^.

To date, current-induced SOT physics is predominantly studied via electrical transport measurements. In metallic magnets, spin–orbit effects have been measured using both (i) direct measurement of current-induced magnetization dynamics^4^,^9^,^10^,^25^, utilizing the non-zero electrical conductivity and the presence of magnetoresistance/anomalous-Hall (AH) effects; and (ii) its Onsager reciprocal process of dynamic magnetization-induced spin/charge pumping^5^,^8^,^13^,^26^. On the other hand, although the reciprocal spin pumping has been observed in insulators^15^,^27^,^28^,^29^,^30^, with virtually zero conductivity, the direct quantitative electrical measurement of SOTs in such materials has proven a challenging task^21^,^31^,^32^,^33^.

Light interacts with the magnetic order of both metallic and insulating materials through the magneto-optical (MO) Kerr effect. In particular, the linear and nonlinear dynamics of the magnetization in virtually any direction, and with high spatial and time resolution, can be studied by employing various microscopy and sub-picosecond pump–probe techniques^34^,^35^,^36^,^37^,^38^,^39^,^40^,^41^. To date, however, very limited efforts, specifically only on selected metallic structures, have been performed to partially incorporate the strength of MO measurements for investigating current-induced dynamics in magnetic heterostructures^42^,^43^,^44^. In particular, the nonlinear MO response of SOT has not been utilized in previous works.

Here we exploit MO measurements to directly probe the spin–orbit fields (SOFs) in two contrasting material systems, one a metallic thin film stack and the other an insulating magnetic heterostructure. The equivalency of MO and transport measurements is established by investigating SOFs of a micrometre-size ultra-thin Ta/CoFeB/MgO device wherein an excellent agreement between the optical and transport methods is found. In contrast to the metallic structures, the SOFs of a 50-nm-thick magnetic insulator yttrium iron garnet (YIG; Y_3_Fe_5_O_12_), in contact with 4-nm-thick Pt, are then directly measured by optical means wherein analogous transport measurements on YIG/Pt are shown to be dominated by other phenomena such as spin-Seebeck effect. Unlike the perpendicular magnetization of the metallic stack, the YIG/Pt structure exhibits in-plane (IP) magnetization. Moreover, we find that both current-induced IP and out-of-plane (OOP) low-frequency oscillations of the magnetization are optically accessible through nonlinear MO terms, and can be separated by tuning the polarization of the incident light. The revealed polarization response of SOTs is unique to the optical measurements, with no analogous counterpart in transport measurements. We quantify a relatively large anti-damping field with a magnitude of 2.89 × 10^−7^ Oe A^−1^ cm^2^ in YIG/Pt, which suggests its potential for spintronic devices based on magnetic insulators. Our results provide a direct and quantitative measurement of SOTs in insulating systems.

## Results

### Experimental set-up and theoretical considerations

**Figure 1:**
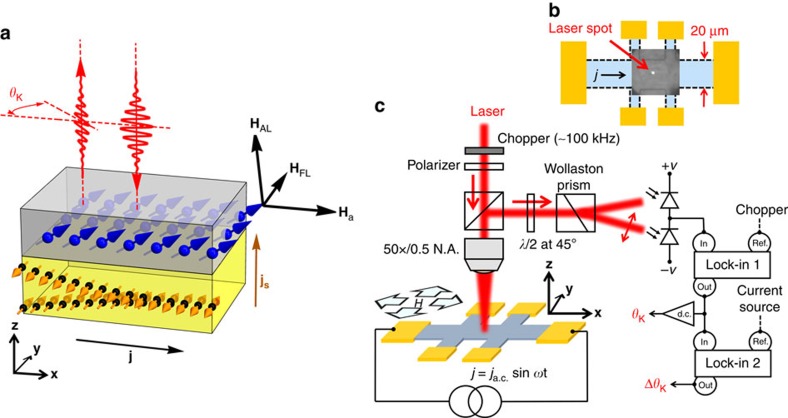
MO probe of SOFs. (**a**) Schematic of the current-induced magnetization dynamics by SOFs, which is directly investigated for both metallic and insulating magnetic structures through the interaction of light with the magnetic order. The direction of applied and SOFs are shown by black arrows. *H*_a_, *H*_FL_ and *H*_AL_ represent applied, field-like and anti-damping-like fields, respectively. Owing to spin–orbit interaction in heavy metal, a lateral current *j* produces a spin current *j*_s_ which propagates in perpendicular direction. (**b**) The optical microscope image of the central region of the device in which the laser (white spot) is tightly focused near the centre of the device. The laser spot size is measured at ∼1 μm, which is much smaller than the dimensions of the device, implying the imaging capabilities of the optical probe. (**c**) Schematic of the experimental set-up depicting the IP current and magnetic field and the backscattering geometry of the probe laser beam. A linearly polarized light is focused on the device using a microscope objective. The intensity of the light is modulated by a photoelastic modulator at 100 kHz. The polarization of the reflected beam is analysed using a half-wave plate at 45°, Wollaston prism and balanced photodiode. Two successive lock-in amplifiers are used to measure the Kerr rotation *θ*_K_ and the modulated Kerr signal Δ*θ*_K_ induced by an IP a.c. current *j* with frequency of ∼277 Hz while the external magnetic field is IP.

The experimental set-up is schematically shown in Fig. 1. In short, a linearly polarized laser beam is tightly focused at the centre of a 20 μm × 130 μm Hall bar device. The measured laser spot is ∼1 μm, much smaller than the dimensions of the device. With an IP applied magnetic field, the magnetization at the laser spot is probed through the MO Kerr rotation (*θ*_K_). The dynamics are induced through the SOTs via an IP a.c. current *j*=*j*_a.c._sin *ωt* while, at the same time, the adiabatic current-induced change of the magnetization (Δ*θ*_K_) at the laser spot is measured. In this backscattering geometry, the Kerr angle is linearly proportional to the OOP component of the magnetization, while the IP magnetization contributes through a second-order term, which is sensitive to the polarization of the incident light. Thus, *θ*_K_ is given by (Supplementary Note 1)


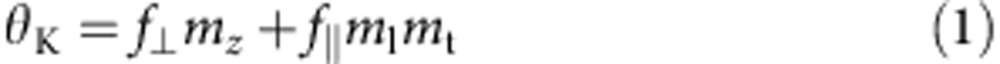


where *m*_*z*_ is the OOP magnetization, *m*_l_ and *m*_t_ are longitudinal and transverse components of the IP magnetization with respect to the polarization of the light, and *f*_⊥_ and *f*_||_ are the first- and second-order MO coefficients that parameterize the strength of the coupling of the light to the OOP and the IP magnetization^35^,^45^,^46^,^47^.

The current-induced magnetization dynamics can be described by the Landau–Lifshitz–Gilbert equation given by


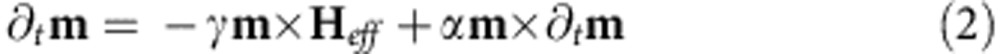


where *m*=*M*/*M*_s_ is the magnetization unit vector normalized to the spontaneous magnetization *M*_s_, *γ* is the gyromagnetic constant and *α* parameterizes the damping. The effective field *H*_eff_ is given by


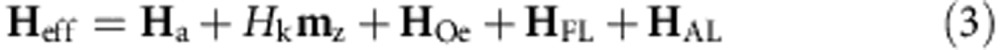


where **H**_a_ is the applied external magnetic field, *H*_k_ is the effective perpendicular anisotropy field and **H**_Oe_ is the current-induced Oersted field. The last two terms are the SOFs, namely field-like (FL) and anti-damping-like (AL) components, with *H*_FL_=*λ*_FL_**z** × **j** and **H**_AL_=*λ*_AL_(**j** × **z**) × **m** (ref. 9). Here, **j** is the current density, **z** is the unit vector normal to the plane and *λ*'s quantify the strength of the SOFs. Since the low-frequency-current-induced dynamics (with frequency of ∼10^3^Hz) are orders of magnitude slower than the magnetization precession frequency (∼10^9^ Hz), it is reasonable to assume that the magnetization adiabatically follows the **H**_eff_ and thus the quasi-equilibrium condition is described by **m** × **H**_eff_=0. Furthermore, we treat the SOT-induced low-frequency oscillation of the magnetization as a perturbation on the equilibrium condition defined by *j*=0.

### SOFs in metallic Ta/CoFeB/MgO

To validate the optical probe, first we investigate an ultra-thin metallic stack of Ta(5 nm)/Co_20_Fe_60_B_20_(1.1 nm)/MgO(2.0 nm) with perpendicular magnetic anisotropy. The advantage of the metallic example is that the optical measurements can be directly compared and correlated with standard transport methods. The device is in a single domain state at the applied bias field and shows current-induced switching that is locally probed at the laser spot (Supplementary Note 2 and Supplementary Fig. 1).

**Figure 2:**
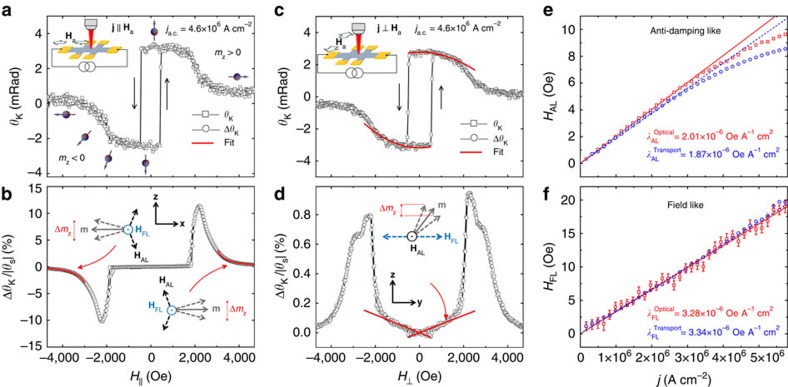
Optical measurements of metallic Ta/CoFeB/MgO. Experimentally measured Kerr angle (*θ*_K_) and (normalized) differential Kerr angle (Δ*θ*_K_) for the magnetic field parallel (**a**,**b**) or perpendicular (**c**,**d**) to the current with current density of 4.6 × 10^6^ A cm^−2^ and with *θ*_S_=*θ*_K_(*H*_a_=0). The schematics in **a**,**b** show the direction of the magnetic field with respect to the current. The arrow balls in **a** schematically show the direction of the magnetic moment at various magnetic field. The *θ*_K_ and Δ*θ*_K_ are proportional to *m*_*z*_ and Δ*m*_*z*_, respectively. The sharp edges in **a**,**c** indicate OOP switching of the magnetization due to a small OOP component of the external field. The asymmetric and symmetric line shapes in **b**,**d** reflect the symmetries of the anti-damping-like and field-like spin–orbit effective fields under magnetization reversal, respectively. The current-induced dynamics of magnetization **m** and relevant directions of SOFs are schematically demonstrated in **b**,**d**. For **j** ||**H**_a_, the anti-damping field is quantified by fitting equation (4) to the experiment at large fields (solid red lines in **b**). For **j**⊥**H**_a_, the field-like effective field is measured from the curvature of *θ*_K_ (solid red line in **c**) and the slope of Δ*θ*_K_ (solid red line in **d**). Comparison of optical (red box) and transport (blue circle) measurements of anti-damping-like and field-like effective fields at various current densities for Ta/CoFeB/MgO is shown in **e**,**f**, respectively. In **e**,**f**, the solid red lines are linear fits to the optical probe, whereas the dashed blue lines are linear fits to the transport results. In **e**, the fits quantify the anti-damping-like coefficients *λ*_AL_ at (2.01±0.01) × 10^−6^ and (1.87±0.01) × 10^−6^ Oe A^−1^ cm^2^ for optical and transport probes, respectively. In **f**, the field-like coefficients *λ*_FL_ for the optical and transport probes are measured at (3.28±0.03) × 10^−6^ and (3.34±0.02) × 10^−6^ Oe A^−1^ cm^2^, respectively. The error bars in **e**,**f** are obtained by linear regression. The error bars in **e** are smaller than symbols. The consistency demonstrates the equivalency and relevance of the MO probe technique for investigating the SOTs in magnetic structures.

With our geometry, the optical measurements on Ta/CoFeB/MgO are dominantly sensitive to the change of the OOP component of the magnetization, with only a minor contribution from the IP oscillations. Figure 2a,b show *θ*_K_ (∼*m*_*z*_) and Δ*θ*_K_ (∼Δ*m*_*z*_) for the magnetic field parallel to the current density of 4.6 × 10^6^ A cm^−2^. At fields larger than *H*_k_, the magnetization is aligned with the external field, which is evident by a nearly constant *θ*_K_≈0. Thus, while *H*_AL_ induces OOP oscillations, *H*_FL_ drives the IP oscillations of the magnetization. The differential Kerr signal (Δ*θ*_K_) is therefore dominantly driven by *H*_AL_ and is relatively insensitive to *H*_FL_. The signal at large fields is proportional to the strength of *H*_AL_, with a 1/(*H*_a_−*H*_k_) dependence indicating the oscillatory nature of Δ*θ*_K_. At near-zero field, Δ*θ*_K_ weakens significantly since the OOP magnetization results in a nearly zero net change of *m*_*z*_ at the first harmonic of the current. At fields larger than the anisotropy, *H*_AL_ can be quantified by (Supplementary Note 1)


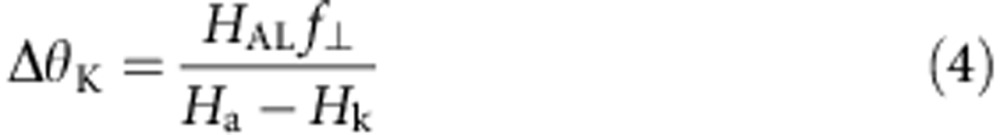


where *f*_⊥_(=*θ*_S_) is readily available from *θ*_K_ at *H*_a_=0, resulting in *H*_AL_=8.50±0.08 Oe for this example. Similarly, *H*_FL_ can be investigated by aligning *H*_a_ perpendicular to the current (Fig. 2c,d). In this case, at low fields, *H*_FL_ oscillates the magnetization in the *y*–*z* plane while the *H*_AL_ causes an IP oscillation, which does not contribute to Δ*θ*_K_. For the current density of 4.6 × 10^6^ A cm^−2^ shown in Fig. 2c,d, the FL effective field is measured at *H*_FL_=14.9±0.7 Oe (see Supplementary Note 3 for details).

The current dependence of *H*_AL_ and *H*_FL_ is summarized in Fig. 2e,f and are compared with the second-harmonic analysis of the AH voltage on the same device. The details of the transport measurements and comparisons are presented in Supplementary Note 3 and Supplementary Figs 2 and 3. For both optical and transport measurements shown in Fig. 2e, while the *H*_AL_ shows a linear dependence on the current densities up to ∼3.5 × 10^6^ A cm^−2^, at larger current a nonlinearity emerges that could be either due to Joule heating or deviation of the dynamics from the linear regime. Linear fits to the lower side of the data yields 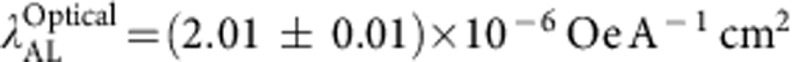 and 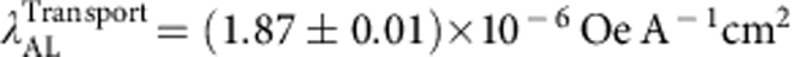
for the optical and transport measurements, respectively. These values, both the magnitudes and the signs, are in agreement with that reported in the literature for a similar structure^9^. The optically measured *λ*_AL_ is ∼7% larger than that of the transport measurement, which may be due to the contribution of the planar-Hall effect and/or other nonlinear terms to the AH resistance in the transport or higher-order terms in the optical measurements. The measured FL coefficients are 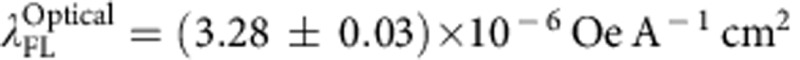 and 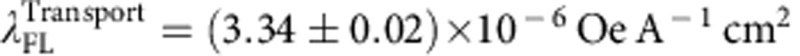, consistent with a previous report^9^.

To summarize this part, the consistency of the MO and transport measurements of SOFs in metallic Ta/CoFeB/MgO establishes both the equivalency and the relevance of the optical probe for investigating SOT-related phenomena.

### SOTs in insulating YIG/Pt

**Figure 3:**
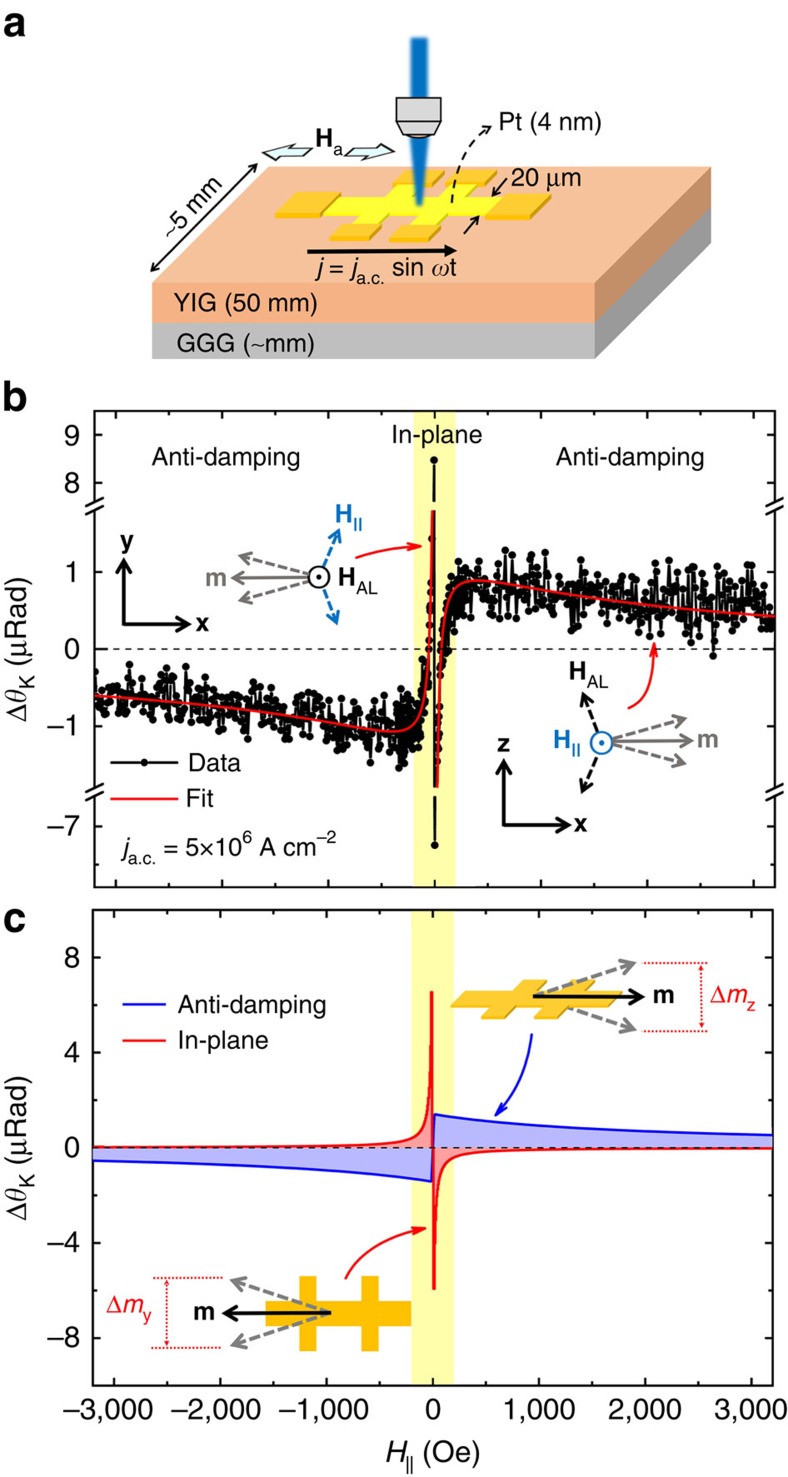
Current-induced differential Kerr of YIG/Pt structure. (**a**) The structure of the material in which a micrometre-size 4-nm-thick Pt device is fabricated on a mm-size 50-nm-thick insulating YIG substrate. (**b**) The differential Kerr signal of YIG/Pt with the magnetic field and the polarization of the incident light parallel to the current. The horizontal dashed line marks Δ*θ*_K_=0. As demonstrated schematically in **b**,**c**, with this geometry, the anti-damping field drives the OOP low-frequency oscillation of the magnetization, whereas the field-like and Oersted fields drive the IP oscillation. The shaded area highlights the sharp diverging-like features at low fields corresponding to the IP oscillations partially driven by the field-like effective field. The broader component, most evident at higher fields, corresponds to the OOP oscillation driven by the anti-damping field. Equation (5) fits well to the experimental data (solid red line in **b**) where the individual contributions due to anti-damping and IP fields are shown in **c**.

Light interacts with the magnetic order of both metallic and insulating magnetic materials. To this end, we use the MO probe to examine a prototypical magnetic-insulator/heavy-metal structure in which the magnetization of the insulator (YIG) is modulated by an IP current through the heavy metal (Pt). As shown in Fig. 3a, the structure consists of micrometre-size 4-nm-thick Pt Hall bar device on a mm-size 50-nm-thick YIG film grown on a gadolinium gallium garnet (GGG) substrate (see Methods, Supplementary Note 4, Supplementary Fig. 4 and refs 23, 48 for more details on YIG). Furthermore, the YIG exhibits an IP magnetization in contrast to the OOP magnetization of Ta/CoFeB/MgO. The details of the measurements are similar to the metallic case. Owing to the IP magnetization, the *θ*_K_ remains constant (zero), whereas a pronounced current-induced Δ*θ*_K_ is observed. An example of Δ*θ*_K_ with the current density *j*_a.c._=5 × 10^6^ A cm^−2^ is shown in Fig. 3b, wherein both the current and the polarization of the laser are parallel to the magnetic field. Interestingly, Δ*θ*_K_ behaves very differently compared with the metallic case. Moreover, two distinct regimes are identified: a sharp diverging-like feature at lower fields and a broader, slow-decaying component most evident at the higher fields. With this geometry, *H*_AL_ and *H*_FL_ point along the OOP and IP directions, respectively. Thus, the differential Kerr signal induced by the current parallel to the magnetic field reads (Supplementary Note 1)


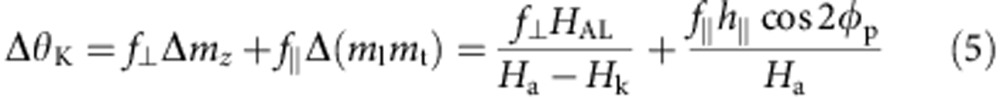


where *H*_k_<0 (unlike *H*_k_>0 in Ta/CoFeB/MgO), *φ*_p_ is the angle between the current and the polarization of the laser, and *h*_||_=*H*_FL_+*H*_Oe_. Both *H*_AL_ and *H*_FL_ contribute to Δ*θ*_K_. Furthermore, the low-frequency OOP oscillation is induced by *H*_AL_ and competes against the *H*_k_ with a 1/(*H*_a_−*H*_k_) dependence, while the free IP oscillation is partly driven by *H*_FL_ and diverges at *H*_a_=0. Comparing with the experimental data in Fig. 3b, we find that the diverging-like and the slow-decaying components are associated with the current-induced IP and OOP oscillations, respectively. The experimental data fit very well to equation (5) using the individual contributions of the IP and OOP oscillations that are reported in Fig. 3c.

**Figure 4:**
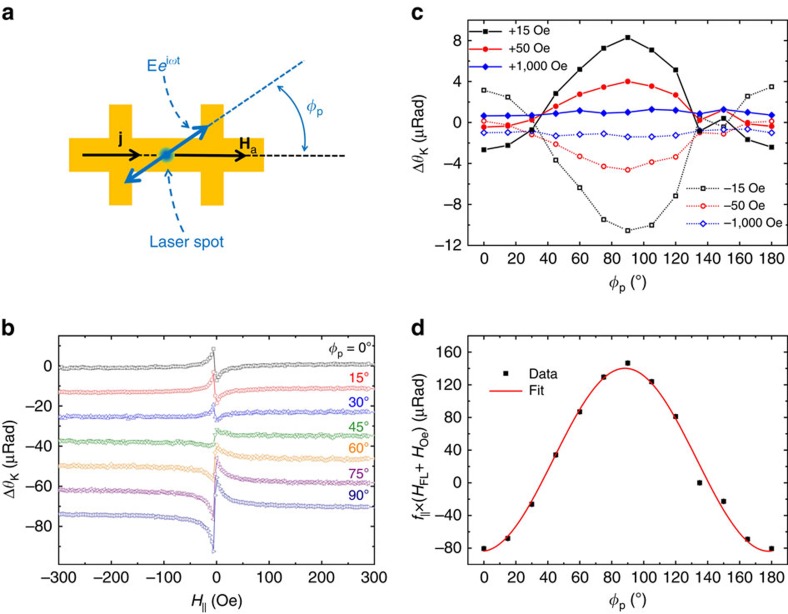
Polarization dependence of the differential Kerr of YIG/Pt. (**a**) Schematic of the measurements in which the IP magnetic field is parallel to the current and the polarization of the perpendicularly incident light (*φ*_p_) is varied with respect to the field. (**b**) Comparison of the differential Kerr signal of YIG/Pt at lower fields for different polarizations of the light. (**c**) Polarization dependence of Δ*θ*_K_ at various external fields. While the differential Kerr signal shows a sinusoidal dependence at low fields, it is insensitive to the polarization at higher fields. (**d**) Amplitude of Δ*θ*_K_ due to IP oscillations extracted from a theoretical fit of equation (5) to the experiment. The solid line in **d** is a theoretical fit to cos 2*φ*_p_. The contribution of the IP oscillation to Δ*θ*_K_ vanishes at *φ*_p_≈40°.

It is noted that the differential Kerr of the IP oscillation is sensitive to the polarization of the incident light, whereas the AL component is insensitive to the polarization; as is verified experimentally. This polarization dependence is unique to the MO probe and has no analogous counterpart in transport measurements. Figure 4 summarizes the polarization dependence of Δ*θ*_K_ at a given current density. The diverging-like component, corresponding to the IP reorientation, shows a strong polarization dependence with minimum and maximum amplitudes at *φ*_p_=0° and 90°, respectively. On the other hand, the Δ*θ*_K_ at higher fields, corresponding to the *H*_AL_-induced OOP oscillations, shows no obvious polarization dependence as illustrated in Fig. 4c. The relative amplitude of the *h*_||_ versus polarization is extracted from a theoretical fit of equation (5) to the experimental data in Fig. 4b and is plotted in Fig. 4d. The data fit well to cos 2*φ*_p_ as predicted by equation (5). The small shift in vertical direction might be due to possible IP anisotropy or higher-order effects that are ignored here. These observations strongly support the attribution of the OOP and IP oscillations to the slow-decaying and the diverging-like components of Δ*θ*_K_, respectively. Although in principle it is possible to extract the value of the *H*_FL_, here however we expect that the *H*_Oe_ dominates the IP oscillation (Supplementary Note 5 and Supplementary Fig. 5). Furthermore, it is noted that the contribution of the IP oscillation is completely suppressed at *φ*_p_≈40° and thus, at this polarization the signal is dominantly induced by the anti-damping field.

**Figure 5:**
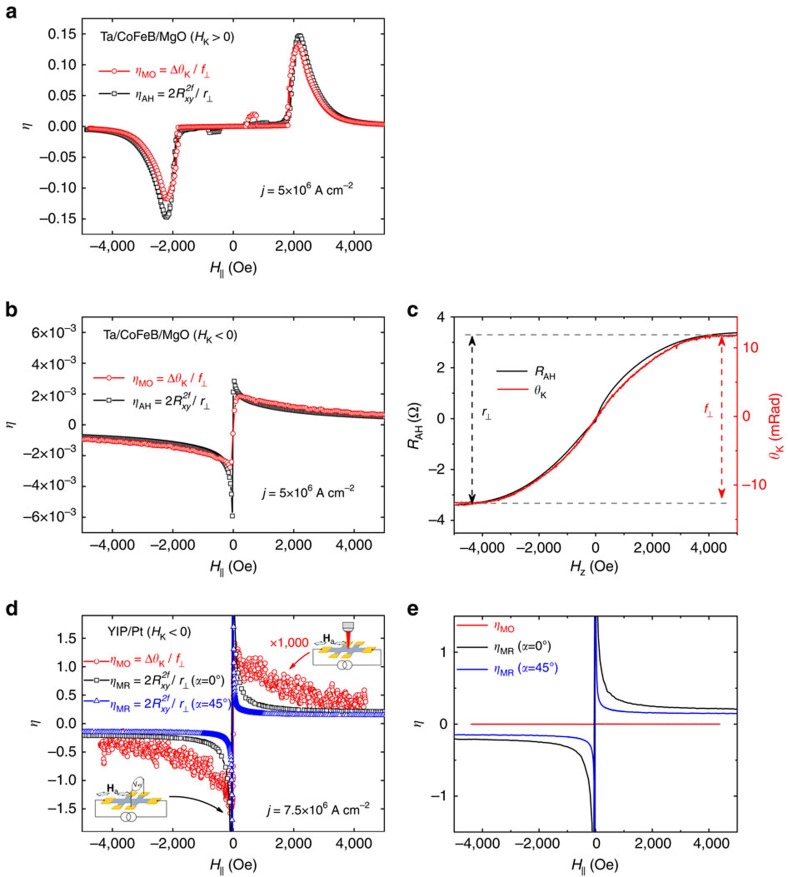
Comparison of MO and transport measurements. Comparison of *η*_MO_=Δ*θ*_K_/*f*_⊥_ from MO (red circles) and 
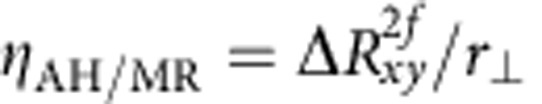
 from second-harmonic anomalous-Hall/transverse-Hall (black boxes) measurements for **a** metallic Ta/CoFeB/MgO with OOP anisotropy (*H*_k_>0), (**b**) Ta/CoFeB/MgO with IP anisotropy (*H*_k_<0) and (**d**) insulating YIG/Pt. In **a**, *f*_⊥_ and *r*_⊥_ are readily available from MOKE/AH measurements. For IP Ta/CoFeB/MgO in **b**,**c** shows MOKE and AH measurements with OOP field to extract *f*_⊥_ and *r*_⊥_, respectively. In **a**,**b** the current is parallel to the field while in **c** *η*_MR_ is demonstrated for both current being parallel (black boxes) and at 45° to the field (blue triangles) corresponding to *α*=0° and 45°, respectively. In **d** *η*_MO_ is expanded by × 1,000 for clarity while the direct comparison of *η*'s is shown in **e**. Equation (6) is validated for Ta/CoFeB/MgO regardless of the sign of the anisotropy as shown in **a**,**b** demonstrating that both optical and transport signals in Ta/CoFeB/MgO originate in SOT. In a sharp contrast the equation (6) is violated for YIG/Pt system as demonstrated in **d**,**e**. In YIG/Pt, the contribution of SOT in transport measurements is significantly overwhelmed by other nonlinear effects (for example, spin-Seebeck effect) resulting in *η*_MR_≫*η*_MO_ as illustrated in **d**. This comparison demonstrates the superiority of MO measurements for studying SOTs in insulating systems.

In a sharp contrast to the metallic case, the harmonic analysis of the transverse-Hall magnetoresistance of YIG/Pt^19^,^32^,^49^,^50^ system is significantly dominated by other nonlinear effects, for example, the spin-Seebeck effect^51^. To demonstrate this, it is instructive to define a dimensionless quantity *η* that relates the MO and transport measurements to the magnetization dynamics through the identity


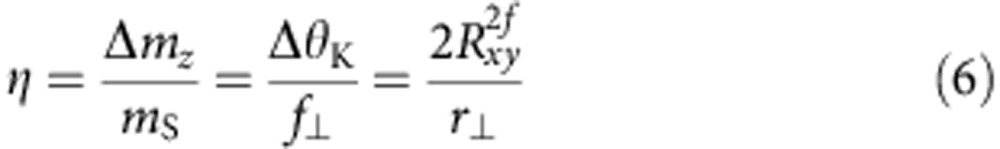


where 
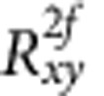
 is the second-harmonic transverse-Hall resistance *R*_*xy*_=*r*_⊥_*m*_*z*_+r_||_*m*_*x*_*m*_*y*_ where the coefficients *r*_⊥_ and *r*_||_ depend on intrinsic material properties (refs 18, 51, Supplementary Note 6 and Supplementary Fig. 6). Noting that the identity (equation (6)) is also valid for the anomalous Hall (AH) effect (Supplementary Note 3), Fig. 5a,b compares *η*_MO_ and *η*_AH_ for Ta/CoFeB/MgO with the OOP and IP anisotropy (*H*_k_>0 and *H*_k_<0, respectively) wherein the values of *η*_MO_ and *η*_AH_ are extracted from either MO or AH measurements, respectively. The coefficients *r*_⊥_ and *f*_⊥_ for the IP Ta/CoFeB/MgO are separately measured by applying magnetic field normal to the plane and are demonstrated in Fig. 5c. Note here that the current is parallel to *H*_a_ resulting in the dominant contribution of the *H*_AL_. In both cases, the identity in equation (6) is verified, which indicates that both the optical and transport signals in Ta/CoFeB/MgO originate in the SOT, regardless of the sign of *H*_k_. In sharp contrast, for the YIG/Pt device the identity (equation (6)) is violated showing *η*_MR_≫*η*_MO_, as illustrated in Fig. 5d, where *η*_MO_ is expanded by 1,000 × for clarity while a direct comparison is presented in Fig. 5e. The measured *r*_⊥_ and *f*_⊥_ that are used to obtain *η*'s for the YIG/Pt device are presented in Supplementary Notes 6 and 7 and Supplementary Fig. 6. In Fig. 5d,e, *η*_MR_ of YIG/Pt is demonstrated for both the current direction being parallel and at 45° to the field (*α*=0° and 45°, respectively). Thus the contribution of *h*_||_ is minimized for *η*_MR_ with *α*=45° as well as *η*_MO_ (Supplementary Note 6). Furthermore, the *η*'s exhibit different field dependences: while *η*_MO_ approaches zero with a 1/(*H*_a_−*H*_k_) dependence, *η*_MR_ remains finite at large fields (even up to 1 T). In addition, with *α*=45°, the presence of the diverging-like signal is not consistent with current-induced IP reorientation (Supplementary Note 6 and Supplementary Fig. 7). These observations strongly suggest that the contribution of *H*_AL_ to the transverse-Hall signal is significantly overwhelmed by other nonlinear effects such as the spin-Seebeck effect (Supplementary Note 6, Supplementary Fig. 8 and ref. 51) and thus may not provide a clean nor direct measurement of the SOTs. It is noted that such nonlinear effects do not contribute to the optical measurements since Δ*θ*_K_∝*j* while the measured second-harmonic transverse voltage 
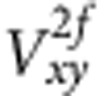
∝*j*^2^.

**Figure 6:**
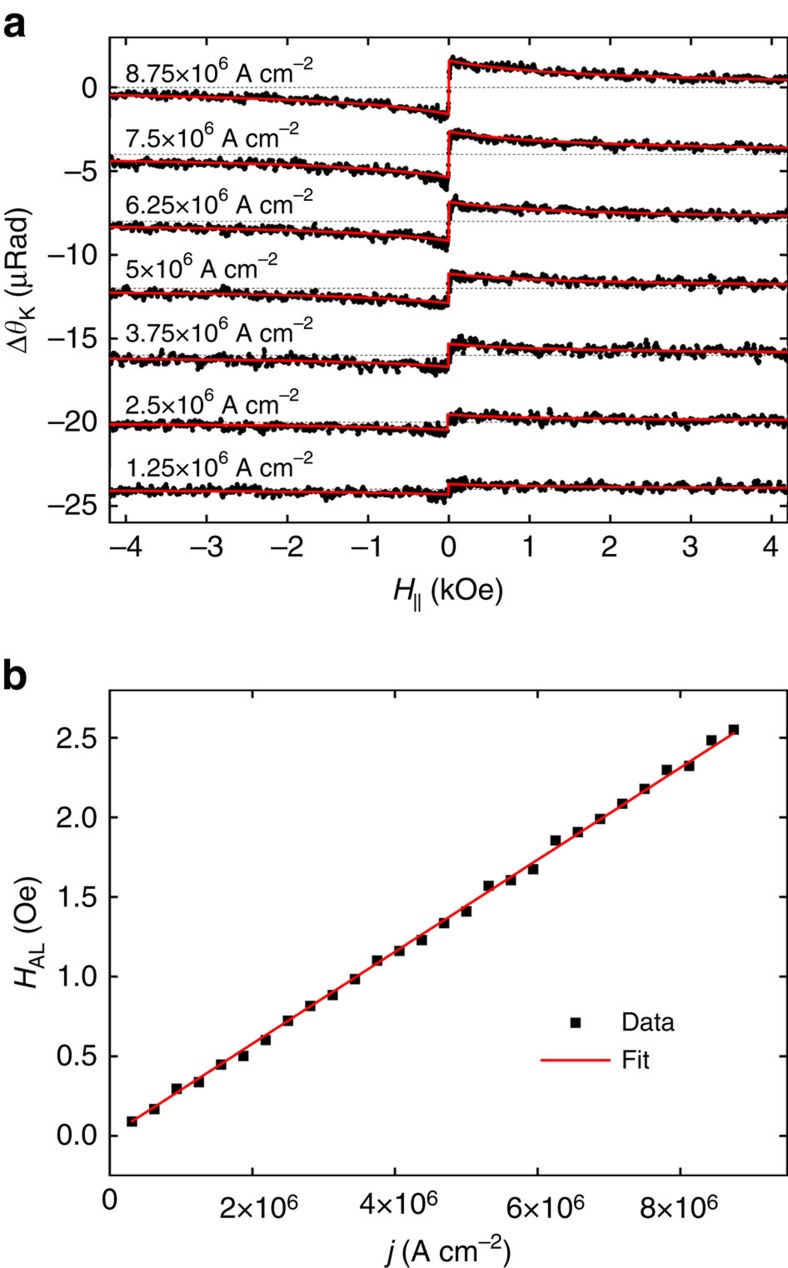
Optically measured anti-damping field of YIG/Pt. (**a**) Current dependence of the differential Kerr signal of YIG/Pt device with the polarization of the light set to *φ*_p_≈40° to minimize the contribution of the IP oscillation. Solid red lines in **a** are theoretical fits of the anti-damping-induced component of equation (6) to the experimental data. Dashed lines in **a** mark Δ*θ*_K_=0. (**b**) The measured anti-damping field in YIG/Pt versus the current density through the Pt device. The error bars in **b** (obtained by linear regression) are smaller than the symbols. The solid red line in **b** is a linear fit to the data that yields the anti-damping coefficient *λ*_AL_=(2.89±0.02) × 10^−7^ Oe A^−1^ cm^2^.

We report the current dependence of Δ*θ*_K_ of YIG/Pt in Fig. 6 with the polarization set to *φ*_p_=40° for which only *H*_AL_ makes a contribution. The data fit very well to the first term of equation (6) providing a quantitative measure of the *H*_AL_. The coefficient *f*_⊥_ in equation (6) is independently measured (Supplementary Note 7 and Supplementary Fig. 9). Figure 6b shows *H*_AL_ at various current densities demonstrating a linear dependence resulting in *λ*_AL_=(2.89±0.02) × 10^−7^ Oe A^−1^ cm^2^ from a linear fit to the data.

## Discussion

In our measurements, the sign of the *λ*_AL_ in YIG/Pt is similar to the positive sign obtained in Ta/CoFeB/MgO. However, in the YIG/Pt, the Pt is on the top of the magnetic structure, whereas in the Ta/CoFeB/MgO the heavy metal (Ta) is at the bottom side of the structure resulting in a sign reversal in each structure with respect to the other. Thus, the *λ*_AL_ in Pt has the opposite sign compared with Ta, which is consistent with their relative signs of the spin-Hall angle^52^. The magnitude of *λ*_AL_ in YIG/Pt is nearly one order of magnitude smaller than that observed for Ta/CoFeB/MgO. One should note however that the 50-nm-thick YIG is substantially thicker than the 1.1-nm-thick CoFeB. Furthermore, the spin transmission efficiency at the YIG/Pt interface could be as small as 0.15 (ref. 21). A more direct comparison can be obtained by noting that *λ*_AL_∼*Tθ*_SH_/(*t*_FM_*M*_S_); where *θ*_SH_ is the spin-Hall angle of the heavy metal, *t*_FM_ is the thickness of the magnetic layer and *T* characterizes the effective spin transmission at the interface of heavy metal and magnetic layer^21^. Using the experimentally measured *λ*_AL_'s for YIG/Pt and Ta/CoFeB/MgO, we obtain (*Tθ*_SH_)_YIG/Pt_/(*Tθ*_SH_)_Ta/CoFeB_=0.69. Here we used values for *M*_s_ of 75 emu cm^−3^ and 700 emu cm^−3^ for YIG and CoFeB, respectively (Supplementary Note 4 and ref. 53). Overall, our data suggest that *λ*_AL_ in YIG/Pt is relatively large and can potentially be used to switch the magnetization by reducing the thickness and perhaps the lateral dimensions of the YIG, as well as using materials with higher spin-Hall angle such as topological insulators^25^,^48^.

Because of the experimental limitations inherent in the transport techniques, very limited efforts have been reported to quantify the strength of the SOTs in YIG-based devices. As discussed earlier, the transverse-Hall magnetoresistance is significantly dominated by other nonlinear transport mechanisms such as the spin-Seebeck effect^51^. Magnetic resonance force microscopy has been employed to investigate the mechanical resonance of a magnetic cantilever dipole coupled to a micro-disk of YIG/Pt where a rate of 0.5 Oe mA^−1^∼1.7 × 10^−7^ Oe A^−1 ^cm^2^ change in the linewidth, including inhomogeneous broadening, for 20-nm-thick YIG was reported^21^. Spin pumping at ferromagnetic resonance and spin-Hall magnetoresistance rectification has also recently been used to investigate a mm-size YIG/Pt structure where one can calculate an anti-damping field of 1.8–2.0 × 10^−7^ Oe A^−1^ cm^2^ from the reported results for 55-nm-thick YIG^30^. However, the dominant contribution of the Oersted field and the complex line shape of the resonance signal demand thickness-dependent measurements along with extensive numerical simulations, which thus limits the quantitative measure of the magnitude of the SOT^54^. While our work suggest that spin-Seebeck and other possible nonlinear effects have a dominant contribution, it might be possible to account for these effects in all-electrical resonance measurements. The MO measurements, however, overcome these limitations and provide a superior direct and quantitative probe of the SOTs in virtually any magnetic-insulator structure with diffraction-limited spatial resolution, regardless of thickness and geometry.

In summary, we demonstrate that SOT physics of magnetic heterostructures are directly accessible and can be accurately measured by optical means, regardless of their electrical conductivity. The relevance of the MO probe is established by investigating a metallic Ta/CoFeB/MgO structure, and is then extended to an insulating YIG/Pt structure where the transport techniques are considerably limited. We reveal that in the optical probe, the polarization of the light also carries information on SOTs, whereas there is no analogous counterpart in transport measurements. Our specific result on YIG/Pt quantifies a relatively large anti-damping field of 2.89 × 10^−7^ Oe A^−1^ cm^2^. Our work opens up exciting opportunities in revealing SOT physics, particularly for investigating the spin-transfer mechanisms and spin-wave physics in magnetic insulators as well as magnetization dynamics of devices with internal magnetic textures.

## Methods

### Ta/CoFeB/MgO

Material stacks consisting of Ta(5 nm)/Co_20_Fe_60_B_20_(1.1 nm)/MgO(2.0 nm)/TaO_x_ layers are sputter deposited at room temperature on a thermally oxidized Si/SiO_2_ substrate. The 2 nm MgO is grown by rf-sputtering from an MgO insulator target. The TaO_x_ layer is formed by oxidizing a 1.5-nm Ta layer under an O_2_/Ar plasma for protection. The films are annealed to enhance the perpendicular magnetic anisotropy. Further details can be found in ref. 53.

### YIG/Pt

Yttrium iron garnet (Y_3_Fe_5_O_12_, YIG) films were grown on GGG (Gd_3_Ga_5_O_12_) (111) substrates using pulsed laser deposition (see Supplementary Note 4 and refs 23, 48 for details). The Pt layer of 4 nm thickness was deposited by d.c. magnetron sputtering at room temperature.

### Device fabrication

The films are patterned into 20 × 130-μm Hall bar devices by standard photolithographic and dry etching techniques.

### Optical measurements

The devices are mounted on a custom built XYZ translational stage and a linearly polarized laser beam is tightly focused on the device using a 50 × /0.42 NA (numerical aperture) long working-distance microscope objective. Special care was taken to assure the optical axis is normal to the plane of the sample with better than 1° accuracy, excluding the finite NA of the objective. The laser spot size is measured at ∼1 μm, which is much smaller than the 20 μm width and 130 μm length of the device and is placed at the centre of the device, both in the lateral and longitudinal directions. At the lateral centre of the device the normal component of the Oersted field vanishes and thus *m*_*z*_ is not directly modulated by the Oersted field. At the maximum current density used in our measurements, the IP component of the Oersted field is estimated at <2 Oe. The back-reflected light is collected by the same microscope objective and rotation of the polarization plane is analysed using a Wollaston prism and a balanced silicon photodetector. To improve the signal-to-noise ratio, the intensity of the laser was modulated at ∼100 kHz using a combination of a photoelastic modulator and a linear polarizer. To modulate the magnetization through SOTs, an a.c. current of *j*=*j*_a.c._ sin *ωt* with frequency of ∼277 Hz, variable amplitude and zero d.c. offset is used. Two successive lock-in amplifiers were employed to analyse the output signal of the balanced photodetector. While the first lock-in (time constant of 100 μs), locked to the frequency of the photoelastic modulator, measures the relative magnitude of the Kerr angle *θ*_K_, the second lock-in (time constant of 300 ms) is locked to the frequency of the current source and probes any change in the Kerr angle induced by the current (Δ*θ*_K_). It should be noted that nonlinear components in Δ*θ*_K_, such as heating, may appear at higher harmonics and thus makes a minor contribution to our first-harmonic measurements. The external magnetic field is kept IP with some small OOP component (<2°) such that *m*_*z*_>0 for positive IP fields. The presented data for Ta/CoFeB/MgO are obtained by employing a 80MHz mode-locked Ti:Sapphire laser centred at 840 nm. The same results are reproduced by 632.8- and 730-nm CW lasers. For YIG/Pt structure, to improve the transmission of the laser through the Pt, a laser beam of 420 nm is employed, which was generated through second-harmonic generation by a beta barium borate crystal pumped by a 840-nm mode-locked laser. This significantly improved the signal-to-noise ratio compared with 840-nm mode-locked or CW lasers. For both Ta/CoFeB/MgO and YIG/Pt, the signal is linearly proportional to the intensity of the laser with no obvious laser-induced heating effects. The presented data are for a laser average intensity of ∼20 μW cm^−2^ for both the metallic and insulating cases. Measurements are performed at ambient condition.

### Transport measurements

Transport measurements are performed immediately after the optical measurement without altering the geometry and with the laser beam being blocked.

## Additional information

**How to cite this article:** Montazeri, M. *et al.* Magneto-optical investigation of spin–orbit torques in metallic and insulating magnetic heterostructures. *Nat. Commun.* 6:8958 doi: 10.1038/ncomms9958 (2015).

## Supplementary Material

Supplementary Information Supplementary Figures 1-9, Supplementary Notes 1-7 and Supplementary References
