# Supplementary material for: Magneto-optical investigation of spin–orbit torques in metallic and insulating magnetic heterostructures
Source: Nat Commun. 2015 Dec 8;6:8958. doi: 10.1038/ncomms9958 (PMC4686864; doi:10.1038/ncomms9958)
Supplement: Supplementary Information — Supplementary Figures 1-9, Supplementary Notes 1-7 and Supplementary References [file ncomms9958-s1.pdf]

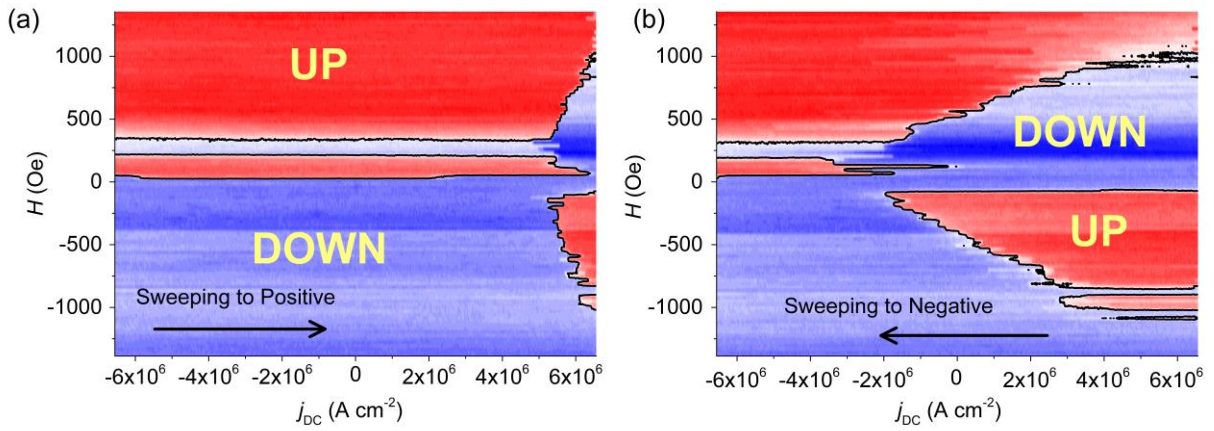


**Supplementary Figure 1. Optically probed current induced switching of Ta/CoFeB/MgO.** The magnetization reversal induced by SOT at the ~1 μm laser spot of the device measured by Kerr angle for **a** positive and **b** negative sweeping of the DC current. The magnetic field is parallel to the current direction. The irregular switching at near zero fields may originate in the multi-domain structure and domain wall motion within the device.

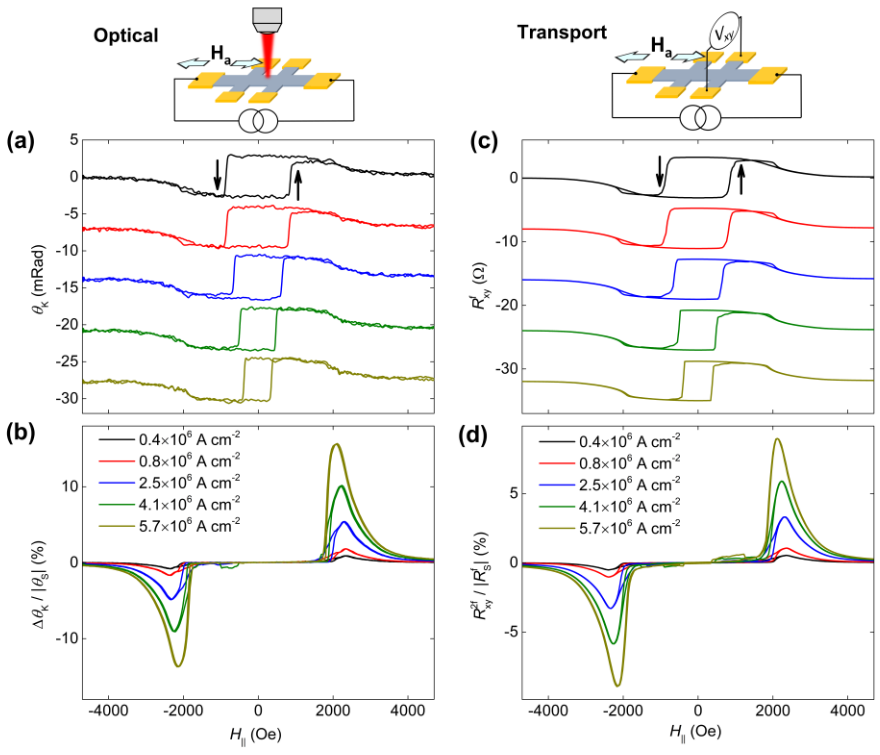


**Supplementary Figure 2. Current dependence and comparison with transport for j ∥ H. a,** Kerr angle ($\theta_K$) and **b,** differential Kerr angle ($\Delta\theta_K$) correspond to **c,** the first-($R_{xy}^{f}$) and **d,** the second ($R_{xy}^{2f}$) harmonic of the Hall resistance respectively. The in-plane magnetic field is parallel to the current. In this geometry, $\Delta m_z$ is driven by the anti-damping field. The reduction of the coercivity in **a** and **c** may indicate Joule heating or large SO fields at higher current densities. The consistency of the optical and transport measurements reflects the equivalency of the two measurements. Details can be found in Supplementary Note 3.

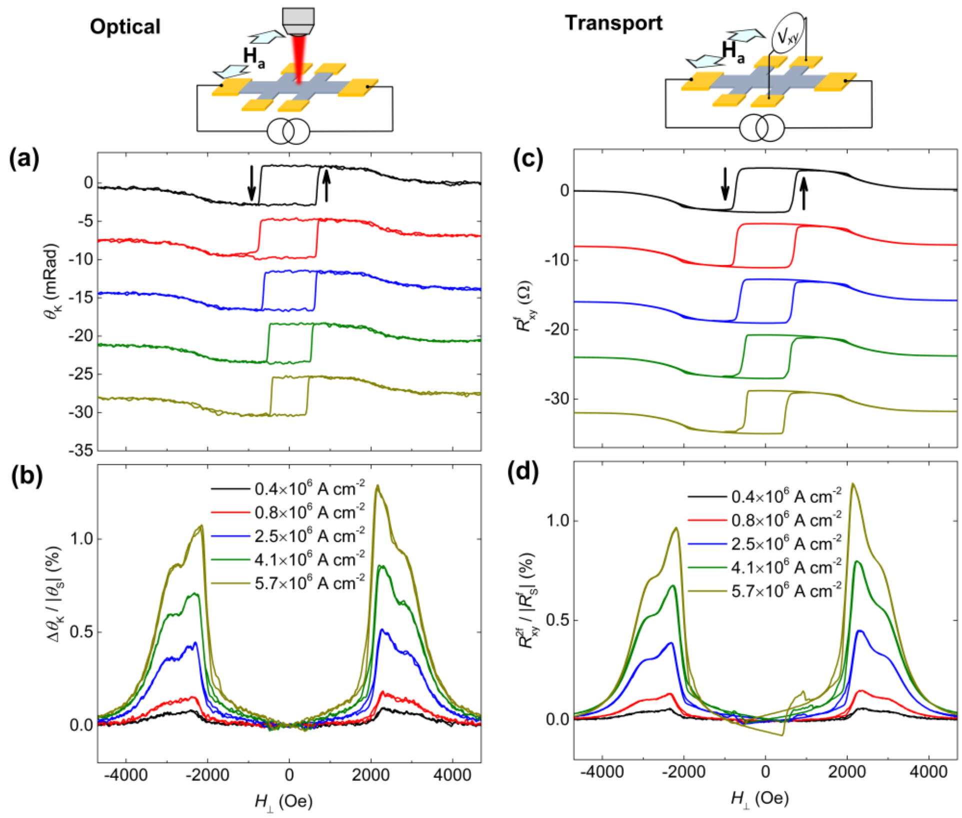


**Supplementary Figure 3. Current dependence and comparison with transport for j ⊥ H. a,** Kerr angle ($\theta_{\mathrm{K}}$) and **b,** differential Kerr ($\Delta\theta_{\mathrm{K}}$) correspond to **c,** the first ($R_{\mathrm{xy}}^{\mathrm{f}}$) and **d,** the second ($R_{\mathrm{xy}}^{2\mathrm{f}}$) harmonic of the Hall resistance, respectively. The in-plane magnetic field is perpendicular to the current. In this geometry, $\Delta m_{\mathrm{z}}$ is driven by the field-like effective field.

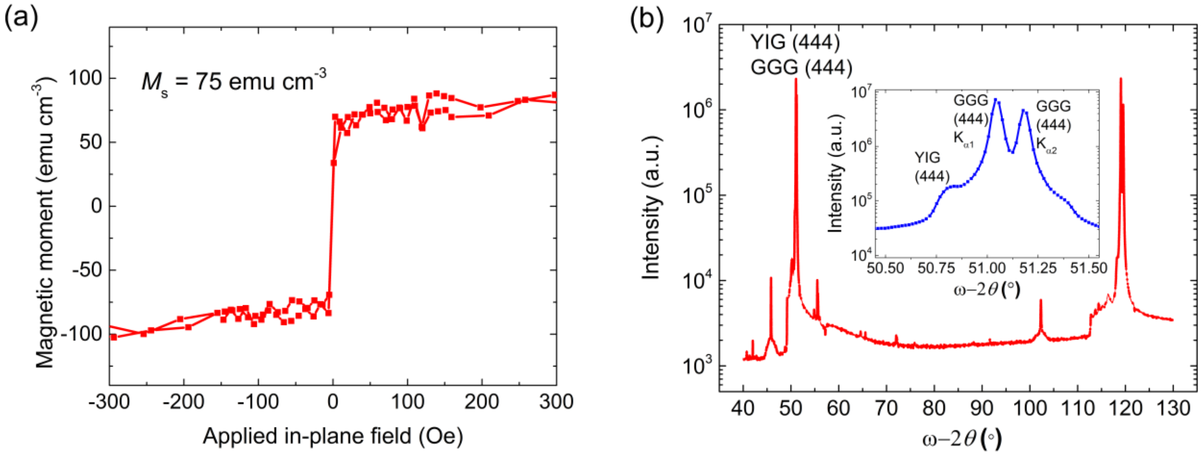


**Supplementary Figure 4. Characterization of YIG/GGG. a**, Magnetic hysteretic loop at 300 K with in-plane magnetic field measured by VSM indicating the in-plane saturation at ~75 emu $\mathrm{cm}^{-3}$ and the coercivity less than 5 Oe. **b**, X-ray diffraction (XRD) spectrum of YIG grown on GGG in $\omega - 2\theta$ scan in which the peaks of the film and the substrate were aligned due to epitaxial growth. **Inset**: expanded angle spectrum indicating (111) orientation of the YIG.

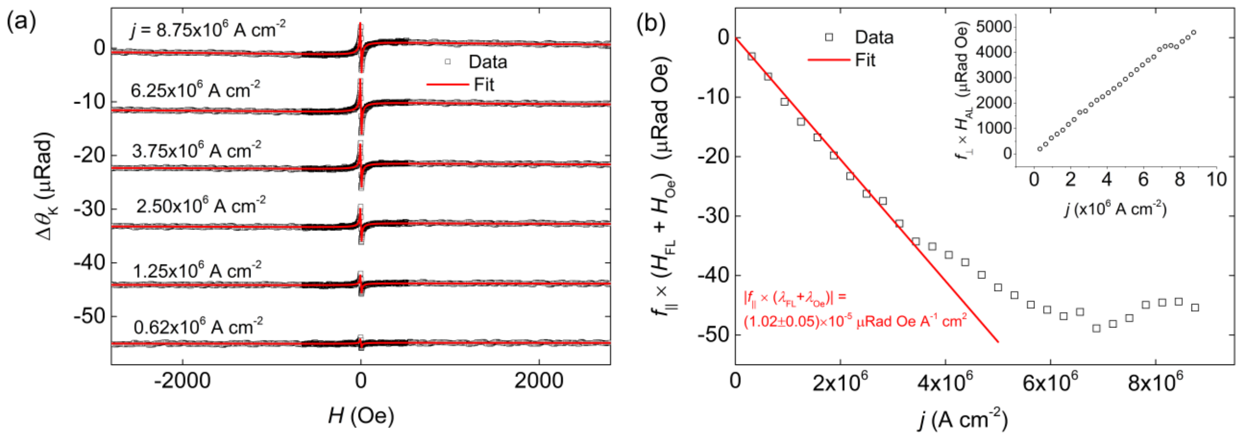


**Supplementary Figure 5. In-plane field in YIG/Pt. a**, The differential Kerr rotation of YIG/Pt device at various current densities where in the polarization of the laser is parallel to the magnetic field ($\phi_p = 0$). In **a**, the diverging-like features at small fields correspond to the in-plane reorientation induced by the current-induced in-plane field $h_\parallel$. The solid red line in **a** is theoretical fit of equation (5) of the text to the data. **b**, Amplitude of the in-plane modulation of the magnetization extracted from the theoretical fit of equation (5) to the experimental data in **a**. The solid-red line in **b** is linear fit to the data which quantifies $|f_\parallel(\lambda_{FL} + \lambda_{Oe})| = (1.02 \pm 0.05) \times 10^{-5}$ μRad Oe A$^{-1}$cm$^2$. The inset in **b** shows $f_\perp \times H_{AL}$ extracted from the theoretical fits shown in **a**.

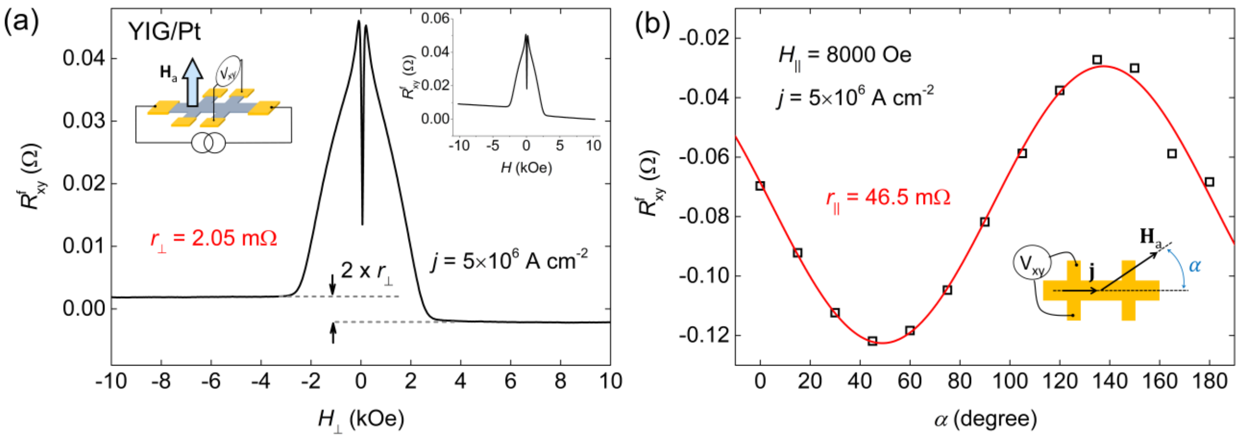


**Supplementary Figure 6. Spin-Hall magneto resistance of YIG/Pt. a**, First harmonic transverse-Hall magnetoresistance of YIG/Pt device with an ac current density of $5 \times 10^6$ A cm$^{-2}$ and magnetic field perpendicular to the device plane. In **a**, the linear background (due to ordinary Hall effect) is subtracted from the raw data shown in the inset. At large fields, the resistance saturates at two values which yields $r_\perp = 2.05$ mΩ. **b**, angle dependence of first harmonic transverse-Hall magnetoresistance of the YIG/Pt device at a current density of $5 \times 10^6$ A cm$^{-2}$ and in-plane magnetic field of $H_\parallel = 8000$ Oe. The solid red line in **b** is a theoretical fit of $\sin 2\alpha$ to the data which yields $r_\parallel = 46.5$ mΩ (see Supplementary Equation 8).

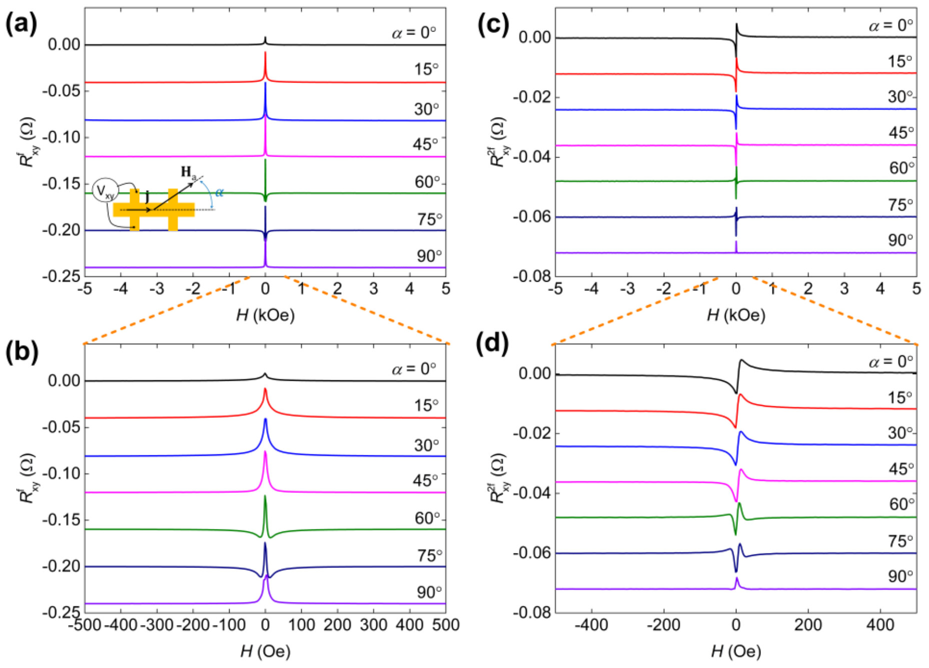


**Supplementary Figure 7. Angle dependence of transverse-Hall magneto-resistance of YIG/Pt.** The first harmonic (**a** and **b**) and the second harmonic (**c** and **d**) of the transverse-Hall magnetoresistance of YIG/Pt device with an ac current density of $5 \times 10^{5}$ A cm$^{-2}$ and in-plane magnetic field at various angles of the magnetic field with respect to the current. **b** and **d** are the low field expansion of **a** and **c**, respectively.

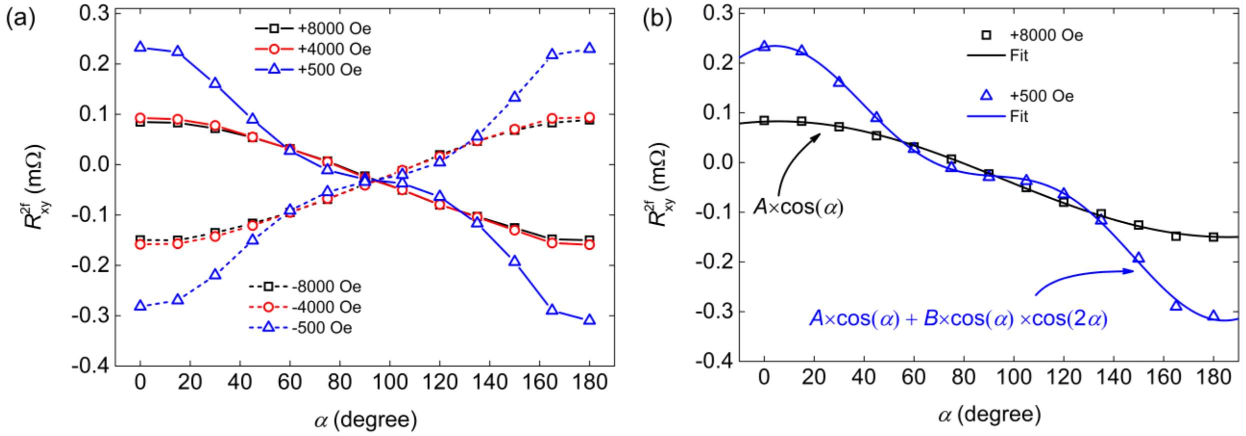


**Supplementary Figure 8. Angle dependence of the second harmonic measurement of YIG/Pt. a**, Angle dependence of second harmonic signal at various magnetic fields. Consistent with the spin-Seebeck effect, it is evident from **a** that at large fields the second harmonic signal saturates to a relatively large finite value. **b**, Theoretical fit to the angle dependence at 8000 and 500 Oe shown by solid black and blue lines, respectively. At large fields, the data fits well with $\cos\alpha$ while at smaller fields $A\cos\alpha + B\cos\alpha\cos 2\alpha$ is used (see text). The current density in **a** and **b** is $5 \times 10^{6}$ A cm$^{-2}$.

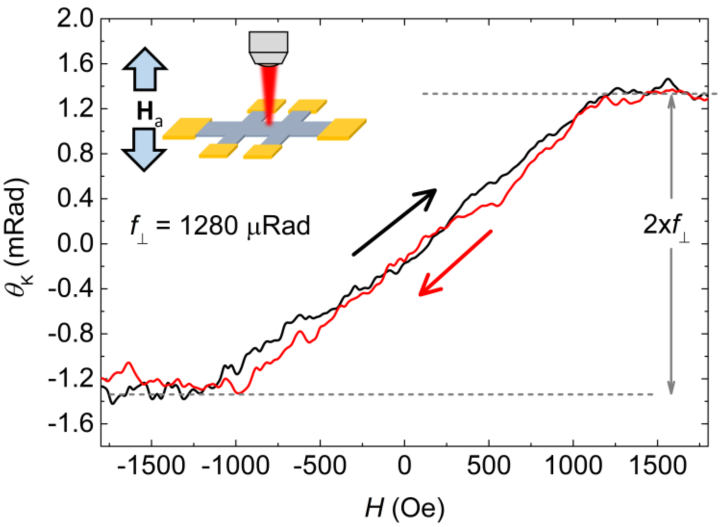


**Supplementary Figure 9. Polar MOKE of YIG/Pt.** The Kerr rotation of YIG/Pt with the applied magnetic field perpendicular to the device plane. The Kerr rotation saturates to $\sim$1280 μRad at $\sim \pm$ 1200 Oe and gives $f_{\perp} = 1280$ μRad.

## Supplementary Note 1. Derivation of Kerr signal induced by spin-orbit torques

In this section we derive the expressions used to extract the current-induced spin-orbit fields via Kerr rotation. As explained in the main text, this is achieved experimentally by exciting the magnetization by an ac current density $j$, at frequency $\omega$, much lower than the ferromagnetic resonance, while simultaneously measuring the Kerr rotation $\theta_{\mathrm{K}}$, at $\omega$ and dc. To this end, taking $j = j_{\mathrm{AC}} \sin \omega t$, the Kerr rotation can be Taylor expanded as:

$$\theta_{\mathrm{K}}(j) = \theta_{\mathrm{K}0} + j_{\mathrm{AC}} \left( \frac{\partial \theta_{\mathrm{K}}}{\partial j} \right) \sin \omega t \tag{1}$$

The information about spin-orbit fields is encoded in $\partial \theta_{\mathrm{K}} / \partial j$, which is caused by the ac spin-orbit field-induced magnetization oscillation translating into an oscillating Kerr rotation at

$\omega$. The relation between the Kerr rotation and magnetization can, in turn, be obtained by measuring the dc Kerr rotation, $\theta_{\mathrm{K0}}$. Thus, the simultaneous measurement of the Kerr signal at $\omega$ and dc suffices to measure the strength of the spin-orbit fields.

The schematic of the experimental setup along with the associated coordinate system is shown in Figure 1 of the main text. All the experiments are performed for near normal (to the film plane) incidence of the laser beam with its axis of polarization making an angle $\phi_{\mathrm{p}}$ with the current (which is oriented along the $x$ axis). Two kinds of magnetic films are studied here, i.e., with either out of plane or easy plane anisotropy. To obtain the required expressions for $\theta_{\mathrm{K0}}$ and $\partial\theta_{\mathrm{K}}/\partial j$ for such geometries, we begin by writing the Kerr rotation in terms of the magnetization, as dictated by the combination of Onsager and structural symmetries [1–4]. The magnetic films considered in this work, in turn, have the following symmetry properties: the inversion symmetry along the axis normal to the film plane is broken due to the presence of different materials on top and bottom of the magnetic film, while the structure is isotropic under rotation about the $z$ axis. The latter condition is fulfilled by the amorphous and/or polycrystalline nature of the films. With these symmetry properties we can write [1–4]:

$$\theta_{\mathrm{K}} = f_{\perp} m_{\mathrm{z}} + f_{\parallel} m_{\mathrm{l}} m_{\mathrm{t}} \tag{2}$$

Here, $\mathbf{m} \equiv (m_{\mathrm{x}}, m_{\mathrm{y}}, m_{\mathrm{z}})$ is a unit vector oriented along the magnetization with the out-of-plane component denoted by $m_{\mathrm{z}}$, while the in-plane components are represented by $m_{\mathrm{l}} \equiv m_{\mathrm{x}} \cos\phi_{\mathrm{p}} + m_{\mathrm{y}} \sin\phi_{\mathrm{p}}$ and $m_{\mathrm{t}} \equiv -m_{\mathrm{x}} \sin\phi_{\mathrm{p}} + m_{\mathrm{y}} \cos\phi_{\mathrm{p}}$. The subscripts l and t stand for longitudinal (along the laser's polarization axis) and transverse (perpendicular to laser's polarization axis) components. Finally, $f_{\perp}$ and $f_{\parallel}$ parameterizes the strength of the so called first and second-order magneto-optic effects [1–4], respectively. As will become clear later, inclusion of

second order effects is crucial for explaining the observed signals, in particular for the films with easy-plane anisotropy. At this point we also note that deviation from the condition of isotropicity can give rise to other second order terms, of the form $\sim m_{\mathrm{l}}^2, m_{\mathrm{t}}^2$, which can be easily included in the presented formalism if demanded by the experiment. We see from Supplementary Equation (2) that $\theta_{\mathrm{K0}}$ can be obtained by substituting the equilibrium magnetization components in the absence of the current, denoted by $\mathbf{m_0}$. While the task of finding the ac component $\partial\theta_{\mathrm{K}}/\partial j$ is reduced to obtaining the deviations from $\mathbf{m_0}$ due to the current, as captured by $\partial m_{\mathrm{z}}/\partial j$, $\partial m_{\mathrm{l}}/\partial j$ and $\partial m_{\mathrm{t}}/\partial j$ evaluated at equilibrium and in the absence of the current, which we carry out next.

When the frequency of excitation is much lower than the ferromagnetic resonance frequency, the magnetization orientation in the presence of the ac current follows the quasi-equilibrium orientation obtained by equating the net torque to zero, i.e., $\mathbf{m} \times \mathbf{H}_{\mathrm{eff}} = 0$. The effective field can be written as:

$$\mathbf{H}_{\mathrm{eff}} = \mathbf{H}_{\mathrm{a}} + H_{\mathrm{k}} m_{\mathrm{z}} \mathbf{z} + \mathbf{H}_{\mathrm{SO}} + H_{\mathrm{Oe}} \mathbf{y} \tag{3}$$

where $\mathbf{H}_{\mathrm{a}}$ is the externally applied magnetic field, $H_{\mathrm{k}}$ parameterizes the strength of effective perpendicular anisotropy (with $H_{\mathrm{k}} > 0$ and $H_{\mathrm{k}} < 0$ for out-of-plane and easy plane anisotropy, respectively), $H_{\mathrm{Oe}} \equiv \lambda_{\mathrm{Oe}} j$ is the net current-induced Oersted field and $\mathbf{H}_{\mathrm{SO}} \equiv \lambda_{\mathrm{AL}} j \mathbf{m} \times \mathbf{y} + \lambda_{\mathrm{FL}} j \mathbf{y}$ represents the current-induced spin-orbit field. This spin-orbit field is comprised of the so called anti-damping-like (AL) and field-like (FL) terms[5] which are expanded up to the first order in the current with the proportionality constants denoted by $\lambda_{\mathrm{AL}}$ and $\lambda_{\mathrm{FL}}$, respectively. Moreover, the range of $j$ is such that these current-induced effective fields are small enough, resulting in small deviations from equilibrium, i.e., each component of $j_{\mathrm{AC}} \partial\mathbf{m}/\partial j \ll 1$. In this case, the condition for the net torque to be zero can be re-expressed in terms of deviations as:

$$\frac{\partial \mathbf{m}}{\partial j} \times \mathbf{H_0} + \mathbf{m_0} \times \frac{\partial \mathbf{H}_{\text{eff}}}{\partial j} = 0 \tag{4}$$

Here, $\mathbf{H_0} = \mathbf{H}_{\text{eff}}(j = 0)$ and $\partial \mathbf{H}_{\text{eff}}/\partial j$ are the effective field contributions corresponding to equilibrium ($j = 0$) and deviations from the equilibrium orientation, respectively. The solution for the case with a general configuration of the applied field is discussed in Ref. [5]; however, in this section we discuss the simple limits used experimentally to extract spin-orbit fields for both out-of-plane and easy plane magnets:

**a. Out-of-plane magnet, i.e., $H_\text{k} > 0$, with $\mathbf{H}_\text{a} = H_\text{a}\boldsymbol{x}$ and $H_\text{a} > H_\text{k}$**

In this case, the equilibrium magnetization in the absence of current is oriented along the $x$ axis, i.e., $\mathbf{m_0} = (1{,}0{,}0)$ and $\mathbf{H}_\text{a} = (H_\text{a}, 0{,}0)$. While the deviations in the field, up to first order in current-induced fields, can be written from Supplementary Equation (3) as $\partial \mathbf{H_{eff}}/\partial j = H_\text{k}(\partial m_\text{z}/\partial j)\mathbf{z} + \lambda_{\text{AL}}\mathbf{m_0} \times \mathbf{y} + (\lambda_{\text{FL}} + \lambda_{\text{Oe}})\mathbf{y}$. Solving Supplementary Equation (4) with above the substitutions and using Supplementary Equation (2) we get:

$$\frac{\partial \theta_\text{K}}{\partial j} = \frac{f_\perp \lambda_{\text{AL}}}{H_\text{a} - H_\text{k}} + \frac{f_\parallel \cos 2\phi_\text{p}(\lambda_{\text{FL}} + \lambda_{\text{Oe}})}{H_\text{a}} \tag{5}$$

Noting that $f_\parallel \ll f_\perp$ (remembering that $f_\parallel$ and $f_\perp$ characterizes the second-order and first-order magneto-optic coefficients, respectively) and $1/(H_\text{a} - H_\text{k}) \gg 1/H_\text{a}$ we can safely neglect the second term on the right hand side of Supplementary Equation (5) for the out-of-plane magnets, resulting in the following formula that is used to extract $\lambda_{\text{AL}}$:

$$\frac{\partial \theta_\text{K}}{\partial j} \approx \frac{f_\perp \lambda_{\text{AL}}}{H_\text{a} - H_\text{k}} \tag{6}$$

**b. Easy plane magnet, i.e., $H_\text{k} < 0$, with $\mathbf{H_a} = H_a\mathbf{x}$**

For this scenario, the relevant expression obtained in Supplementary Equation (5) is still valid. The difference from the out-of-plane magnets is that the expression is valid for arbitrary field strength (as long as the magnet remains single domain) and, more importantly, since now $H_{\mathrm{k}} < 0$, we have $1/(H_{\mathrm{a}} - H_{\mathrm{k}}) \ll 1/H_{\mathrm{a}}$. Consequently, we can no longer neglect the second term on the right hand side of Supplementary Equation (5). In fact, at low fields the signal may be dominated by the second term, which is expected to show a dependence on the polarization axis with respect to the current, as observed experimentally. This polarization dependence is used to extract and separate the contribution of the anti-damping-like term from the field-like and Oersted fields, as explained in the main text.

**Supplementary Note 2. Current-induced switching in Ta/CoFeB/MgO**

In addition to spin-orbit fields, current-induced magnetization reversal can also be probed optically with diffraction limited spatial resolution. To demonstrate this, Supplementary Figure 1 shows the current induced switching of the magnetization of the same device discussed in the text by probing the Kerr angle as a function of a lateral DC current. In this measurement, at each given magnetic field, the current was swept from negative to positive and then from positive to negative. Here, the magnetic field is parallel to the current and thus the anti-damping-like torque initiates the magnetization reversal. The ability to study the dynamics of current-induced switching with high spatial resolution could be useful in investigating the dynamics of domain walls and the internal magnetic textures of nanodevices.

**Supplementary Note 3. Current dependence of Ta/CoFeB/MgO and comparison with transport measurements**

The current-induced dynamics of the magnetization due to $H_{\mathrm{FL}}$ are studied by making the in-plane external magnetic field perpendicular to the current as described in the text. In this case, at low fields, the $H_{\mathrm{FL}}$ oscillates the magnetization in the $\mathbf{y}-\mathbf{z}$ plane while the $H_{\mathrm{AL}}$ causes an in-plane oscillation which does not contribute to $\Delta\theta_{\mathrm{K}}$. As demonstrated in the Fig. 2c and d of the text, while the Kerr rotation is similar to the parallel case (Fig. 2a) as expected, $\Delta\theta_{\mathrm{K}}$ exhibits a symmetric line shape consistent with the symmetry of the $H_{\mathrm{FL}}$ under magnetization reversal. Noting the dominant contribution of the FL field to the OOP oscillations at lower fields, it is more trivial to obtain the $H_{\mathrm{FL}}$ by examining $\theta_{\mathrm{K}}$ and $\Delta\theta_{\mathrm{K}}$ at small applied fields by[6,7]

$$H_{\mathrm{FL}} = -\frac{2\,\partial(\Delta\theta_{\mathrm{K}})/\partial H_{\mathrm{a}}}{\partial^2\theta_{\mathrm{K}}/\partial H_{\mathrm{a}}^2} \tag{7}$$

The solid red lines in Fig. 2c and d of the text show the parabolic and linear fits to extract the curvature of $\theta_{\mathrm{K}}$ and the slope of $\Delta\theta_{\mathrm{K}}$ resulting in $H_{\mathrm{FL}} = 14.9 \pm 0.7$ Oe for this example. The additional uncertainty in the optically probed $H_{\mathrm{FL}}$ presented in Fig. 2f of the text is due to the uncertainty in estimating the curvature of the Kerr angle at lower fields.

The dependency of the optical measurements of Ta/CoFeB/MgO on the amplitude of the AC current is shown in Supplementary Figure 2a and b. We have observed a reduction of the coercivity with increasing the current amplitude which may suggest increasing of the structure's temperature by Joule heating or large spin-orbit fields at higher current densities. However, the modulated signal ($\Delta\theta_{\mathrm{K}}$) increases linearly with the current and the line shape remains consistent. It is noted that the out-of-plane component of the Oersted field vanishes at the laser spot (center of the device). Nevertheless, $H_{\mathrm{Oe}}$ is estimated to be $< 2$ Oe for the maximum current density used here.

The metallic nature of Ta/CoFeB/MgO allows a direct comparison of the optical and standard transport measurements of spin-orbit fields. In order to check the consistency with transport measurements, we have performed both anomalous Hall (AH) measurements at the first-($R_{\mathrm{xy}}^{\mathrm{f}}$) and the second harmonic ($R_{\mathrm{xy}}^{2\mathrm{f}}$) on the same device immediately after optical measurements and with the laser beam being blocked on the device. Details of the transport technique and analysis are discussed in [5,6,8]. The spin-orbit fields can be extracted from AH measurements using the expression $R_{\mathrm{xy}}^{2\mathrm{f}}/R_{\mathrm{xy}}^{\mathrm{f}} = \Delta m_{\mathrm{z}}/2\ m_{\mathrm{S}}$ where $m_{\mathrm{S}}$ is the out-of-plane magnetization at $H_{\mathrm{a}} = 0$. The comparison of the optical and transport measurements and their dependence on the current density are shown in Supplementary Figure 2a-d for the magnetic field being parallel to the current. In this geometry the $\Delta m_{\mathrm{z}}$ is driven by the anti-damping effective field.

The results of the optical and transport measurements are in excellent agreement. Note that the ratio $R_{\mathrm{xy}}^{2\mathrm{f}}/R_{\mathrm{S}}^{\mathrm{f}}$ is nearly half of the ratio of the differential Kerr, which is consistent with $\Delta m_{\mathrm{z}}/m_{\mathrm{S}} = \Delta\theta_{\mathrm{K}}/\theta_{\mathrm{S}} = 2\,R_{\mathrm{xy}}^{2\mathrm{f}}/R_{\mathrm{S}}^{\mathrm{f}}$, where $\theta_{\mathrm{S}}$ and $R_{\mathrm{S}}^{\mathrm{f}}$ are the Kerr angle and AH resistance (first harmonic) at $H_{\mathrm{a}} = 0$, respectively (derived from the data in Supplementary Figure 3a and c). We have used equation (4) of the main text for both optical and transport measurements to extract the current dependency of the AL field.

Similar measurements for the magnetic field perpendicular to the current are shown in Supplementary Figure 3. In this case, $\Delta m_{\mathrm{z}}$ is driven by the field-like effective field. The consistency of both the optical and transport measurements is also evident. Supplementary Equation (7) is used to extract the strength of $H_{\mathrm{FL}}$ for both optical and transport measurements.

The direct comparison and the consistency of optical and transport measurements indicate that, firstly the optical measurements can be used to study SOT, and secondly that the device is very uniform since the AH measurements represent the average magnetization dynamics of the device, whereas the optical measurement samples only an area of a few micrometers.

**Supplementary Note 4. Growth and characterization of YIG**

Yttrium iron garnet ($Y_3Fe_5O_{12}$, YIG) films were grown on Gadolinium Gallium Garnet ($Gd_3Ga_5O_{12}$, GGG) (111) substrates using pulsed laser deposition. 1-inch diameter stoichiometric YIG targets were prepared following the recipe described previously [9,10]. $10$ mm $\times$ $10$ mm GGG (111) substrates (Supplier: MTI Crystals Inc.) were ultrasonicated first in acetone and then in isopropanol for surface cleaning. Next, the substrates were loaded into the chamber and the chamber was pumped down to $5 \times 10^{-6}$ Torr base pressure. After reaching the base pressure at $23°\mathrm{C}$, the oxygen partial pressure has been set to $25$ mTorr. Then, the substrates have been heated under constant oxygen pressure until $650°\mathrm{C}$ substrate temperature and the films were deposited by ablating the YIG target. A KrF excimer laser ($\lambda = 248$ nm, pulse duration $25$ ns, pulse rate $10$ Hz) was used to grow the films. The laser spot size on the target was $0.5 \times 2.0$ mm and the energy density at the target surface was $\sim 1.3$ J cm$^{-2}$ during the pulse. During deposition, the YIG target was rotated to achieve good stoichiometry transfer from the target to thin films. The growth rate was $3$ nm min$^{-1}$ and $50$ nm thick YIG films were grown. After deposition, the chamber was cooled in oxygen ambient at $5$ °C min$^{-1}$ until $200$ °C. The target-substrate distance was kept at $70$ mm for all samples.

Phases inside the films were identified using x-ray diffraction and $\omega - 2\theta$ scans of the films using a PANalytical X'Pert PRO MPD X-ray diffractometer (Cu $K_\alpha$ radiation source at the wavelength of $0.1541$ nm). The samples were scanned with a $1°$ substrate tilt so as to diminish the intensity of the substrate peaks while increasing the film's signal. The films are single-phase and lattice-matched to the substrate. Magnetic hysteresis loops of YIG films were measured at room temperature ($23$ ˚C) using a vibrating sample magnetometer (Digital Measurement Systems Torque/Vibrating Sample Magnetometer Model 1660 Signal Processor, VSM) when the applied magnetic fields were parallel to the sample top surface plane. The paramagnetic contribution of the GGG substrates was removed by subtracting a linear function from the raw magnetic hysteresis loop data. The films had a saturation moment of $M_\mathrm{s} = 75\ \mathrm{emu\ cm^{-3}}$ and coercivity of $H_\mathrm{c} < 2\ \mathrm{Oe}$ (smaller than VSM field step size) and about $0.3$ nm of RMS surface roughness measured using atomic force microscopy (AFM, AFM Nanoscope IV, tapping mode operation at $2$ Hz scan rate) for $50$ nm thick YIG films. A step size of $2\ \mathrm{Oe}$ was used to acquire hysteresis loops for fields below $100\ \mathrm{Oe}$. YIG had an in-plane easy axis due to its dominant shape anisotropy. Although the YIG sample in Supplementary Figure 4 has lower than bulk saturation magnetization, this deposition method has been shown to make films with bulk-like properties[9].

**Supplementary Note 5. Field-like effective field in YIG/Pt**

Because of the in-plane anisotropy of the YIG/Pt system, the current-induced in-plane reorientation (demonstrated by the diverging-like features in Fig. 3 of the text) is driven by an in-plane field with a contribution of both field-like and current induced Oersted fields: $h_\parallel = H_\mathrm{FL} + H_\mathrm{Oe} = j(\lambda_\mathrm{FL} + \lambda_\mathrm{Oe})$. Note that FL and Oersted fields are collinear and, similar to the case of Ta/CoFeB/MgO, the FL term can be separated out if its magnitude is much larger than $H_\mathrm{Oe}$

which is expected to be the case for nm thick YIG systems. The magnitude of the Oersted field for a current density of $j$ can be estimated by $H_{\mathrm{Oe}} = \frac{1}{2} jt$, where $t$ is the thickness of Pt layer[11]. For 4 nm thick Pt (our case) and with a current density of $j = 5 \times 10^{6}$ A cm$^{-2}$, the Oersted field is calculated at $H_{\mathrm{Oe}} = 1.26$ Oe; a value larger than the anti-damping field at the same current density. Noting the rather large thickness of the YIG ($\sim 50$ nm), in contrast to the nm-thick CoFeB, it is expected that the current-induced Oersted field dominates the in-plane reorientation.

Supplementary Figure 5 shows the current dependence of the differential Kerr rotation of the YIG/Pt device. Here, in order to improve the accuracy of the theoretical fit, the polarization of the laser was kept parallel to the magnetic field ($\phi_{\mathrm{p}} = 0$). Note that the broader signal due to the anti-damping torque cannot be suppressed (in a similar manner that was done in Fig. 4 of the main text to investigate the anti-damping field) since it is insensitive to the polarization. The data fits well to equation (5) of the text (solid red lines in Supplementary Figure 5-a) for which $f_{\parallel} h_{\parallel} = f_{\parallel} \times (H_{\mathrm{FL}} + H_{\mathrm{Oe}})$ as well as $f_{\perp} H_{\mathrm{AL}}$ were treated as a free parameters. The values of $f_{\parallel} h_{\parallel}$ at different current densities is reported in Supplementary Figure 5-b. Note that the amplitude of the in-plane modulation deviates from the linear regime at current density of $3 \times 10^{6}$ A cm$^{-2}$ and then saturates to a nearly constant value at densities above $6 \times 10^{6}$ A cm$^{-2}$. At the same time, as shown in the inset of Supplementary Figure 5-b, the amplitude of the out-of-plane modulation ($f_{\perp} H_{\mathrm{AL}}$) remains linear in the entire range. A possible explanation of the saturation of the in-plane reorientation is that at large current densities, $h_{\parallel}$ is sufficiently large to reorient the magnetization (at zero external magnetic field) in the direction of $h_{\parallel}$. As mentioned before, $h_{\parallel}$ is expected to include a dominant contribution from the Oersted field. It is noted that, in the non-linear regime, the theoretical description developed in Supplementary Note 1 based on small linear oscillations of the magnetization may not be valid. A linear fit to the linear region at

smaller current densities quantifies $\left|f_{\parallel} \times (\lambda_{\rm Oe} + \lambda_{\rm FL})\right| = (1.02 \pm 0.05) \times 10^{-5}\ \mu\text{Rad Oe A}^{-1}\ \text{cm}^2$. In order to extract the magnitude of $h_{\parallel}$, one needs to experimentally measure the non-linear magneto-optical coefficient $f_{\parallel}$. From Supplementary Equation (2) and the geometry presented in Fig. 4 of the text, the Kerr rotation reads:

$$\theta_{\rm K}(\phi_{\rm p}) = f_{\perp} m_{\rm z} + \frac{1}{2} f_{\parallel} \sin 2\phi_{\rm p} \tag{8}$$

in which $\phi_{\rm p}$ is the angle between polarization of the laser and magnetization. Supplementary Equation (8) suggests that one can use the polarization dependence of the Kerr rotation to experimentally measure $f_{\parallel}$ to extract the magnitude of the in-plane field. However, our experimental attempts on measuring $f_{\parallel}$ were not conclusive due to the contribution from the polarization response of the optical components that dominate the small non-linear magneto-optical coefficient ($f_{\parallel}$). Considering previous discussion, and noting that $\lambda_{\rm FL}$ is expected to be more than one order of magnitude smaller than $\lambda_{\rm AL}$[12], one can estimate $f_{\parallel} \sim 10\ \mu\text{Rad}$, which is more than two orders of magnitude smaller than the experimentally measured linear coefficient $f_{\perp} = 1280\ \mu\text{Rad}$ (see Supplementary Note 7). Nevertheless, it should be possible to measure $f_{\parallel}$ (and hence $h_{\parallel}$) if material systems have larger non-linear magneto-optical coefficients.

## Supplementary Note 6. Second harmonic analysis of the spin-Hall magnetoresistance of YIG/Pt

Second harmonic analysis of the anomalous Hall voltage has been widely used to characterize spin-orbit torques in metallic magnetic structures[5]. As we have demonstrated in the text and in Supplementary Note 3, we have also used this technique to investigate metallic

Ta/CoFeB/MgO in order to validate the optical measurements for which we have found an excellent agreement. Similar measurements, reported previously, on YIG/Pt suggest a dominant contribution of the spin-Seebeck effect (SSE) in the second harmonic signal of YIG/Pt[11]. In this section we show that, unlike the metallic case, the harmonic analysis of the transverse-Hall magnetoresistance[13] of the YIG/Pt system is significantly dominated by other non-linear effects (e.g. the spin-Seebeck effect) and thus cannot be used to cleanly measure the anti-damping field in this structure. The transverse-Hall magnetoresistance across the YIG/Pt device reads[11,12]:

$$R_{\mathrm{xy}} = r_{\perp} m_{\mathrm{z}} + r_{\parallel} m_{\mathrm{x}} m_{\mathrm{y}} = r_{\perp} m_{\mathrm{z}} + \frac{1}{2} r_{\parallel} \sin 2\alpha \tag{9}$$

wherein $m_{\mathrm{z}}$ is the magnetization perpendicular to the device plane, $m_{\mathrm{x}}$ and $m_{\mathrm{y}}$ are its in-plane components parallel and perpendicular to the current, respectively, and $\alpha$ is the angle between the magnetic field and the current. Proximity-induced magnetoresistance and spin-Hall magnetoresistance are the two possible microscopic mechanisms that have been proposed to give rise to non-zero $r_{\perp}$ and $r_{\parallel}$. In particular, with the spin-Hall magnetoresistance mechanism, the coefficients $r_{\perp}$ and $r_{\parallel}$ depend on the spin-diffusion length, spin-Hall angle and spin-mixing conductance. Note the pronounced similarity of Supplementary Equation (9) with the equation (1) of the main text describing the Kerr rotation. With an ac current through the device, the second harmonic transverse-Hall magnetoresistance is given by:

$$R_{\mathrm{xy}}^{2\mathrm{f}} = \frac{1}{2}\left(\frac{r_{\perp} H_{\mathrm{AL}}}{H_{\mathrm{a}} - H_{\mathrm{k}}} + \frac{r_{\parallel} h_{\parallel} \cos 2\alpha}{H_{\mathrm{a}}}\right) \cos \alpha \tag{10}$$

where $H_{\mathrm{AL}}$ and $h_{\parallel}$ are the anti-damping and in-plane fields with similar definitions described in the text as well as Supplementary Note 1. As discussed in the text, one can define a dimensionless quantity $\eta$ to relate the optical and transport measurements to the magnetization dynamics:

$$\eta = \frac{\Delta m_z}{m_S} = \frac{\Delta\theta_K}{f_\perp} = \frac{2R_{xy}^{2f}}{r_\perp} \tag{11}$$

Indeed, we have verified the identity in Supplementary Equation (11) for Ta/CoFeB/MgO with both in-plane and out-of-plane anisotropy, which was presented in Fig 5a and b of the main text. As discussed in the text, the measurements in Fig. 5a and b are for the case where the magnetic field is parallel to the current with dominant contribution of the anti-damping field. It is noted that the contribution of the in-plane reorientation in the in-plane Ta/CoFeB/MgO is negligible. For the perpendicular device, $r_\perp$ and $f_\perp$ are extracted from the anomalous Hall and Kerr rotation at $H_\parallel = 0$ (see Fig. 2 of the main text). Similarly, for the in-plane device, $r_\perp$ and $f_\perp$ are extracted from the anomalous Hall signal and polar MOKE with the magnetic field perpendicular to the device plane, as illustrated in the inset of Fig. 5b. In both cases, the identity in Supplementary Equation (11) is validated which indicates that both the modulated magneto-optical and the second harmonic anomalous Hall signal originate from SOT.

As discussed in the text and presented in Fig. 5d and e, the Supplementary Equation (11) does not hold for the YIG/Pt system. We find that the contribution of the anti-damping field in the transport measurement is overwhelmed by other non-linear transport effects resulting $\eta_{MR} \gg \eta_{MO}$. First, we experimentally quantify the coefficients $r_\perp$ and $r_\parallel$ in the YIG/Pt device.

Supplementary Figure 6a shows the first harmonic of the transverse-Hall signal of the YIG/Pt device with an ac current density of $j_{AC} = 5 \times 10^6$ A cm$^{-2}$ and with the magnetic field perpendicular to the device plane. In the Supplementary Figure 6, a linear background due to the ordinary Hall effect is subtracted while the inset shows the raw data. The transverse-Hall signal at low fields is evident and similar to previous reports[12]. At larger fields, the transverse-Hall saturates to different values; where the height of the step gives $r_\perp = 2.05$ mΩ. In order to

measure $r_{\parallel}$, a large $(8000\ \mathrm{Oe})$ in-plane magnetic field is applied and the first harmonic transverse-Hall was measured as a function of the angle between the field and current $(\alpha)$. The results are shown in Supplementary Figure 6b. The data fits well with $\sin 2\alpha$ (solid red line in Supplementary Figure 6b) as expected from Supplementary Equation (9) and gives $r_{\parallel} = 46.5\ \mathrm{m}\Omega$. It is important to note that, unlike the magneto-optical response for which $f_{\perp} \gg f_{\parallel}$, in the transverse-Hall $r_{\perp} \ll r_{\parallel}$ which suggests a less pronounced contribution from the linear term in the transport signal (and consequently the anti-damping torque).

Supplementary Figure 7 summarizes the transverse-Hall measurements on YIG/Pt with the in-plane magnetic field at various angles with respect to the current. The experimental data is similar to what was reported earlier. The second harmonic signal, although showing some similarities with the optical measurement, is believed to be dominated by the spin-Seebeck effect rather than SOT[11]. In particular, contrary to what is expected from Supplementary Equation (10), the diverging-like feature at small fields persists at $\alpha = 45°$. Furthermore, by increasing $\alpha$ toward $90°$, additional features appear at low fields which were not observed in the optical measurements and suggest additional contributions to the second harmonic signal. It should be mentioned that our attempts to fit the data with Supplementary Equation (10), even at large fields, has failed or resulted in an unrealistic magnitude of torque and anisotropy field $(H_{\mathrm{k}} \sim 10^{5}\ \mathrm{Oe})$. This suggest that the SOT signal is overwhelmed by other phenomena, including the spin-Seebeck effect. As discussed in the text and presented in Fig 5c, this can be further verified by direct comparison of transport and optical measurements to verify the identity in Supplementary Equation (11), as validated for Ta/CoFe/B in Fig. 5a and b.

Figure 5c of the main text directly compares $\eta_{\mathrm{MO}} = \frac{\Delta\theta_{\mathrm{K}}}{f_{\perp}}$ and $\eta_{\mathrm{MR}} = \frac{2R_{\mathrm{xy}}^{2\mathrm{f}}}{r_{\perp}}$ for the YIG/Pt device with a current density of $7.5 \times 10^{6}$ A cm$^{-2}$. In order to minimize the contribution of the field-like torque in either measurements, the polarization of the laser in the magneto-optical measurement ($\theta_{\mathrm{p}}$) and the direction of the current in transverse-Hall measurement ($\alpha$) are set at $45°$ with respect to the magnetic field. Note that $\alpha = 45°$ introduces a factor of $1/\sqrt{2}$ in Supplementary Equation (10), which is taken into account in Fig. 5d. Similar results are obtained for $\alpha = 0°$ as demonstrated in Fig. 5d. As discussed in the text, in a sharp contrast to the case of Ta/CoFeB/MgO (Fig. 5a and b), for the YIG/Pt device where $\eta_{\mathrm{MR}} \gg \eta_{\mathrm{MO}}$, which strongly suggests that the second harmonic transverse-Hall signal is dominated by other non-linear effects rather than the anti-damping torque. While a direct comparison of the $\eta$'s is shown in the Fig 5e, the $\eta_{\mathrm{MO}}$ in Fig. 5d is expanded by $1000 \times$ for clarity. It is also clear from the figure that the $\eta$'s have different field dependence: while $\eta_{\mathrm{MO}}$ approaches zero with a $1/(H_{\mathrm{a}} - H_{\mathrm{k}})$ dependence, $\eta_{\mathrm{MR}}$ remains finite at large fields, which is consistent with the spin-Seebeck effect. Therefore, while the optical measurement is dominated by the anti-damping field, the contribution of the anti-damping field is significantly overwhelmed by other non-linear effects and thus may not provide a clean and direct measurement of the anti-damping-field. Furthermore, with $\alpha = 45°$, at small fields a sharp diverging-like feature is present, which is not consistent with the current-induced in-plane reorientation (second term in Supplementary Equation (10)) and strongly suggests dominant contributions from other phenomena even at low fields.

In the following we demonstrate that the observed second harmonic transverse magnetoresistance is consistent with the spin-Seebeck effect (SSE) reported previously[11]. The

transverse voltage due to SSE can be written as $V_{\mathrm{T,SSE}} \sim j^2 \cos\alpha$, which is nonlinear with current and sensitive to the direction of the magnetic field. Hence, the SSE contribution to the second harmonic measurement reads:

$$R_{\mathrm{SSE}}^{2\mathrm{f}} = r_{\mathrm{SSE}} \times \cos\alpha \tag{12}$$

where $r_{\mathrm{SSE}}$ is the resistance change depending on the temperature gradient at the interface, thickness of the Pt and other intrinsic properties such as spin-mixing conductance, spin-Hall angle, spin diffusion length, etc[11]. Since the SSE is non-linear with current, it does not contribute to the first harmonic measurements and thus Supplementary Equation (9) is still valid. One should note that the angle dependence of $R_{\mathrm{SSE}}^{2\mathrm{f}}$ is similar to that for the anti-damping field described in Supplementary Equation (10).

Supplementary Figure 8 shows the angle dependence of the second harmonic transverse-Hall signal of YIG/Pt at a current density of $5 \times 10^6$ A cm$^{-2}$ and at various fields. It is evident from Supplementary Figure 8-a that at large fields the maximum magnitude of the second harmonic signal at $\alpha = 0°$ and $180°$saturates to a finite value of $R_{\mathrm{xy}}^{2\mathrm{f}} \sim 0.1 - 0.15$ mΩ. This is in a sharp contrast with the optical measurement (see Fig. 4 of the text) and is consistent with the spin-Seebeck effect. At large fields, the angle dependence fits well with $\cos\alpha$ as expected from Supplementary Equation (12) as clearly shown by a solid black line in Supplementary Figure 8b. On the other hand, additional features appear at fields as small as 500 Oe. In this region, the angle dependence fits well with $A\cos\alpha + B\cos\alpha\cos 2\alpha$ with $A$ and $B$ being free parameters (solid blue line in Supplementary Figure 8b). The $\cos\alpha\cos 2\alpha$ dependence is consistent with the current-induced in-plane reorientation described by the second term of Supplementary Equation (10) and may suggest a contribution of the in-plane reorientation to the second

harmonic signal which might be due to a large $r_{\parallel}$ for YIG/Pt. However, one should note that, as discussed before, other phenomena may contribute at even smaller fields. These observations are all consistent with a previous report on the spin-Seebeck effect in YIG/Pt studied by second harmonic measurements[11].

To summarize this part, we conclude that, in a sharp contrast to the metallic Ta/CoFeB/MgO system (as shown in Fig. 5 of the main text), the optical and transport measurements in YIG/Pt have different origins. This is because the differential magneto-optical measurement is sensitive to linear effects ($\Delta\theta_{\mathrm{K}} \sim j$), whereas the second harmonic measurement is sensitive to nonlinear effects ($V_{\mathrm{xy}}^{2\mathrm{f}} \sim j^2$). Furthermore, the second harmonic measurements for YIG/Pt is dominated by the spin-Seebeck and other possible nonlinear effects, which significantly overwhelm the contribution of the anti-damping field by more than two orders of magnitude. It is important to note that such non-linear effects do not contribute to the differential magneto-optical measurements since $\Delta\theta_{\mathrm{K}} \sim j$.

**Supplementary Note 7. Quantifying $f_{\perp}$ in YIG/Pt**

As explained in the main text, the Kerr rotation is related to the magnetization by $\theta_{\mathrm{K}} = f_{\perp} m_{\mathrm{z}} + f_{\parallel} m_{\mathrm{l}} m_{\mathrm{t}}$. In order to measure the anti-damping-like field of YIG/Pt, the magneto-optical coefficient $f_{\perp}$ needs to be quantified. This is done by measuring the Kerr rotation with the magnetic field perpendicular to the device plane to saturate the magnetization in the $+\mathbf{z}$ and $-\mathbf{z}$ directions (polar MOKE geometry). The coefficient then is given by:

$$\theta_{\mathrm{K}}(+H_{\max}) - \theta_{\mathrm{K}}(-H_{\max}) = 2f_{\perp} \qquad (13)$$

The Kerr rotation of Pt/YIG with the field perpendicular to the plane is shown in Supplementary Figure 9. The Kerr angle saturates at $\sim \pm 1200$ Oe which yields $f_{\perp} =$

1280 μRad. For this measurement, the laser was focused on the same spot of the same device reported in the main text. Similarly, the same wavelength (420 nm) is used to avoid a possible wavelength dependence of $f_{\perp}$.

**Supplementary References**


1. Straub, M., Vollmer, R. & Kirschner, J. Surface Magnetism of Ultrathin γ-Fe Films Investigated by Nonlinear Magneto-optical Kerr Effect. *Phys. Rev. Lett.* **77,** 743–746 (1996).
2. Postava, K. *et al.* Anisotropy of quadratic magneto-optic effects in reflection. *J. Appl. Phys.* **91,** 7293–7295 (2002).
3. Mewes, T., Nembach, H., Rickart, M. & Hillebrands, B. Separation of the first- and second-order contributions in magneto-optic Kerr effect magnetometry of epitaxial FeMn/NiFe bilayers. *J. Appl. Phys.* **95,** 5324–5329 (2004).
4. Hamrle, J. *et al.* Huge quadratic magneto-optical Kerr effect and magnetization reversal in the Co2FeSi Heusler compound. *J. Phys. Appl. Phys.* **40,** 1563–1569 (2007).
5. Garello, K. *et al.* Symmetry and magnitude of spin-orbit torques in ferromagnetic heterostructures. *Nat. Nanotechnol.* **8,** 587–593 (2013).
6. Pi, U. H. *et al.* Tilting of the spin orientation induced by Rashba effect in ferromagnetic metal layer. *Appl. Phys. Lett.* **97,** 162507 (2010).
7. Kim, J. *et al.* Layer thickness dependence of the current-induced effective field vector in Ta|CoFeB|MgO. *Nat. Mater.* **12,** 240–245 (2013).
8. Fan, Y. *et al.* Magnetization switching through giant spin–orbit torque in a magnetically doped topological insulator heterostructure. *Nat. Mater.* **13,** 699–704 (2014).

9. Onbasli, M. C. *et al.* Pulsed laser deposition of epitaxial yttrium iron garnet films with low Gilbert damping and bulk-like magnetization. *APL Mater.* **2,** 106102 (2014).

10. Lang, M. *et al.* Proximity Induced High-Temperature Magnetic Order in Topological Insulator - Ferrimagnetic Insulator Heterostructure. *Nano Lett.* **14,** 3459–3465 (2014).

11. Vlietstra, N. *et al.* Simultaneous detection of the spin-Hall magnetoresistance and the spin-Seebeck effect in platinum and tantalum on yttrium iron garnet. *Phys. Rev. B* **90,** 174436 (2014).

12. Vlietstra, N. *et al.* Exchange magnetic field torques in YIG/Pt bilayers observed by the spin-Hall magnetoresistance. *Appl. Phys. Lett.* **103,** 032401 (2013).

13. Nakayama, H. *et al.* Spin Hall Magnetoresistance Induced by a Nonequilibrium Proximity Effect. *Phys. Rev. Lett.* **110,** 206601 (2013).
